# Hypomethylating therapy mitigates acute allograft rejection in a murine lung transplant model

**DOI:** 10.3389/frtra.2025.1612523

**Published:** 2025-06-23

**Authors:** Kristine M. Yarnoff, William N. Daccarett-Bojanini, Andres F. Villabona-Rueda, Manuel Sollmann, Franco R. D’Alessio, Jeffrey M. Dodd-o

**Affiliations:** ^1^ Department of Anesthesiology and Critical Care Medicine, Johns Hopkins Medical Institution, Baltimore, MD, United States; ^2^ Division of Pulmonary and Critical Care Medicine, Department of Medicine, Johns Hopkins Medical Institution, Baltimore, MD, United States

**Keywords:** lung transplantation, acute rejection, T regulatory cells, decitabine, immune tolerance

## Abstract

**Introduction:**

Acute cellular rejection of transplanted lung allografts involves activated cytotoxic T cells and reduced Regulatory T (Treg) cell function. Calcineurin inhibitors, the cornerstone of immunosuppressive regimens, suppress T cell cytotoxicity but inhibit Treg proliferation. The DNA hypomethylating agent decitabine (DAC) can abrogate T cell cytotoxicity while stimulating Treg proliferation.

**Methods:**

We sought to determine the effects of DAC treatment in a murine MHC-mismatched orthotopic lung transplant model.

**Results:**

Rescue treatment with DAC maintains lung allograft gross and histologic integrity with a reduction in cytotoxic T cell responses. CD4+FoxP3+ T cell depletion in Foxp3DTR mice exacerbated rejection lung injury compared to CD4+FoxP3+ T cell sufficient mice and failed to abolish the protective effect of DAC in this model. The protective effect of DAC was associated with a reduction in cytokine production from host T-cells.

**Discussion:**

Decitabine could offer a new line of treatment for acute lung allograft rejection, in part via its effects on Tregs.

## 1 Introduction

Lung transplantation is the final treatment option for many patients with advanced lung disease. An estimated 28% of lung transplant recipients experience at least one episode of treated acute rejection in the first year following transplantation (1). In acute cellular rejection of transplanted lungs, clonally expanded host CD8 + cytotoxic and effector T cells populate the allograft (2). Regulatory T cells (Tregs) can protect against acute (3) and chronic (4) rejection. However, calcineurin inhibitors, a central component of many immunosuppressive regimens, inhibit Treg proliferation in the setting of allogeneic transplantation (5). This drives the search for a better understanding of, and alternative treatment strategies for, lung transplant rejection.

Decitabine (5-Aza-2'-deoxycytidine) (DAC) is an FDA-approved hypomethylating agent that irreversibly inactivates DNA methyltransferase 1 (DNMT1), thereby inhibiting DNA methylation. Disrupting DNA methylation can impair CD8+ T cell survival (6) and cytolytic activity (7) during live viral infection (lymphocytic choriomeningitis virus), while also promoting the proliferation of immunosuppressive CD4 + Foxp3+ Tregs in cardiac transplantation and diabetes models (8–11). We hypothesize that, by facilitating Treg expansion, DAC administration will interrupt acute cellular rejection to promote allograft tolerance and attenuate the rejection process. Compared to vehicle-treated hosts, DAC treatment beginning post-transplant day 3 reduced graft injury at post-transplant day 10. To evaluate the requirement for resident CD4 + Foxp3+ Tregs, Foxp3DTR mice C57BL/6 recipients receiving diphtheria toxin (dT) or DAC + dT were used. The protective effect of DAC was partially lost in CD4 + FoxP3+ Treg-depleted hosts and was associated with marked decreases in cytotoxic T-cell responses.

## 2 Methods

### 2.1 Mice

Male and female C57BL/6 (H-2b) and BALB/c (H-2d) mice (25–35 g) from Jackson Laboratory (Bar Harbor, ME) and Foxp3DTR mice (background strain C57BL/6, H-2b) originally gifted from Alexander Rudensky (Sloan-Kettering Institute, New York, NY) were bred and housed in a pathogen-free facility before surgery. Open access conditions existed after surgery. All animal protocols were approved by the Johns Hopkins Animal Care and Use Committee.

### 2.2 Lung transplant and pharmacologic administration

Donor left lungs (BALB/c mice) were transplanted into wild-type C57BL/6 or FoxP3DTR using cuffed technique (12). Intraperitoneal DAC (1 mg/kg) (Sigma-Aldrich) or vehicle (DMSO) was administered on days 3, 4, 5, and 8 post-operatively (13). CD4 + FoxP3+ Treg depletion was accomplished by intraperitoneal diphtheria toxin (dT) (List Biologicals, Campbell, CA) injection into Foxp3DTR mice post-implant days 3 (20 ng/kg), 5 (10 ng/kg), and 7 (10 ng/kg). CD4 + Foxp3+ Treg-sufficient mice were instead C57BL/6 wild-type mice receiving intraperitoneal DMSO (with or without dT) post-implant days 3, 5, and 7. Lungs were harvested on day 10 post-implant.

### 2.3 Flow cytometry

Left and right lungs were enzymatically digested separately (13) to generate a single-cell suspension. Cells were prepared for FACS using fluorochrome-conjugated antibodies for surface and intracellular markers (Supplementary S1). A fixable, UV-excitable Blue Dead Cell Stain (Invitrogen) was used for live-dead discrimination, and UltraComp eBeads (eBioscience) were utilized for compensation. Flow cytometry analysis was performed using a FACSAria instrument, and data were analyzed using FlowJo software (Tree Star Inc, San Carlos, CA, USA).

Flow cytometry gating was performed to identify and quantify specific immune cell populations in the allograft (Supplementary S2A and S2B). Events were first gated to exclude debris and doublets, selecting singlets (FSC-W vs. FSC-H) and single cells (SSC-W vs. SSC-H). Live cells were identified using the viability dye and gated accordingly. Lymphocytes were gated based on SSC-A vs. FSC-A, and within this population, CD4 + and CD8+ T cells were selected separately. Further gating was applied to analyze the expression of GATA-3, CTLA-4, FoxP3, CD25, CD44, CD62l, PD-1, CD103, and Ki-67. Tregs were identified as CD4+/FOXP3 + and CD8+/FOXP3 + subsets and analyzed for the same markers. Additionally, within the CD8 + population, we identified Live CD8+/CD44+/CD62l+, Live CD8+/CD103+, and Live CD8+/CD44+/CD62l+/CD103 + subpopulations. Mean fluorescence intensity (MFI) was defined as the geometric mean fluorescence intensity of the positive population.

### 2.4 Host T cell cytokine production

Single-cell suspensions of host responder cells were prepared from host native right lungs harvested 10 days after allograft left lung implantation. After pelleting, cells were resuspended in a pre-warmed R10 medium and incubated overnight for 12 h (37°C, 5% CO2) in an attempt to achieve a rested state. Similarly, right lung was used to obtain a cell suspension free of donor cells which could act as stimulators during the overnight incubation. Stimulator BALB/c spleen cells were similarly prepared, though without the overnight incubation. Responder lung cells and stimulator splenocytes (1:1 ratio, 100,000 cells of each type) were incubated (37°C, 5% CO2) for 5 h with GolgiStop™ Protein Transport Inhibitor (BD Biosciences) present for the final 2 h. Following stimulation, cells were harvested and stained with surface antibodies and intracellular cytokines for flow cytometry analysis. Fluorochrome-conjugated antibodies from BioLegend (FITC anti-H2 kb, AF647 anti H2kq, AF700 anti H2kd, BV650 anti-TNF-α, BV768 anti-CD3), BD Biosciences (PE-CF594 anti-CD19, BV421 anti-IL-17, BUV395 anti-CD4, BUV797 anti-CD8), and ThermoFisher (PE anti-INF-*γ*) were used. Zombie Aqua Fixable Dye (BioLegend) was used for live-dead discrimination.

### 2.5 Histopathology and acute rejection pathology scoring

Grafts were fixed in 10% formalin after harvesting. Embedding (paraffin), sectioning, and staining with Hematoxylin & Eosin were performed by the Reference Histology Core of Johns Hopkins University School of Medicine. Two blinded observers scored stained sections using standard criteria developed by the International Society for Heart and Lung Transplantation (ISHLT) grade A Lung Rejection Study Group (14).

### 2.6 Immunofluorescence staining

Quadruple immunolabeling for CD4 + CD8 + CK19 + CD3 was performed at the Oncology Tissue Services Core of Johns Hopkins University School of Medicine on formalin-fixed, paraffin-embedded (FFPE) sections using a Ventana Discovery Ultra autostainer (Roche Diagnostics). After dewaxing and rehydration, epitope retrieval was performed with Ventana Ultra CC1 buffer (Cat. #6414575001, Roche Diagnostics) at 96°C for 64 min. Immunostaining was performed sequentially for each marker, with individual rounds consisting of primary antibody incubation at 36°C for 40 min, detection using an anti-rabbit HQ detection system (Cat. #7017936001 and #7017812001, Roche Diagnostics), and signal amplification with OPAL fluorophores (Akoya Biosciences) diluted 1:200 in 1X Plus Amplification Diluent (Cat. #FP1498, Akoya Biosciences). For CD8 detection, a rabbit anti-rat linker antibody (1:500; Cat. #AI4001, Vector Labs) was applied at 36°C for 32 min before the HQ detection system. The antibodies used were anti-CD4 (1:200; Cat. #ab133616, Abcam) detected with OPAL 570, anti-CD8 (1:125; Cat. #4SM16, Invitrogen) detected with OPAL 690, anti-CK19 (1:1,000; Cat. #ab133496, Abcam) detected with OPAL 520, and anti-CD3 (1:200; Cat. #16669, Abcam) detected with OPAL Polaris 780. After each round of staining, primary and secondary antibodies were stripped using Ventana Ultra CC1 buffer at 95°C for 12 min, followed by neutralization with Discovery Inhibitor (Cat. #7017944001, Roche Diagnostics). Finally, sections were counterstained with spectral DAPI (Cat. #FP1490, Akoya Biosciences) and mounted with Prolong Gold (Cat. #P36930, ThermoFisher Scientific). Slides were viewed and scanned using the Olympus IX83 Inverted Microscope FISHscope and the Olympus cellSens software. Images were analyzed using ImageJ.

### 2.7 Statistics and visualization

To assess the differential expression of markers on allograft live cells across two treatment groups (DMSO and DAC) and two mouse models (Treg-sufficient and Treg-depleted), a series of pairwise comparisons was performed. We first compared the percentage of live cells expressing each marker between the DMSO and DAC groups within Treg-sufficient mice and repeated this analysis within Treg-depleted mice. We then compared the treatment effects between Treg-sufficient and Treg-depleted mice to evaluate how Treg depletion influenced the response to DAC treatment. Statistical comparisons between two groups were performed using a two-tailed Mann–Whitney (unpaired, non-parametric) test in GraphPad Prism (GraphPad Software, San Diego, CA, USA). Outliers, defined as below Q1–1.5×IQR for low-range outliers and above Q3 + 1.5×IQR for upper-range outliers, were excluded.

For visualization in the volcano plots, log2 fold change (log2FC) was calculated as the log2-transformed ratio of the percentage of allograft live cells expressing each marker between treatment groups. Separate volcano plots were generated using the mean fluorescence intensity (MFI) of each marker, with log2 fold change computed as the ratio of MFI values between treatment conditions. *P*-values for both analyses were obtained from the Mann–Whitney test and transformed using -log10(*p*-value) to represent statistical significance. Markers were classified as upregulated (log2FC > 0, bright blue) or downregulated (log2FC < 0, bright red). Significant markers (*p* < 0.05, -log10 *p*-value > 1.301) were shaded in a green background, while non-significant markers were shaded in gray (Supplementary S2C). All statistical analyses and figure generation, including volcano plots, were performed using GraphPad Prism. This approach allowed visualization of differential expression both in terms of the percentage of marker-positive cells and the intensity of marker expression on individual cells, providing complementary insights into the immunological shifts in the allograft.

## 3 Results

### 3.1 DAC treatment preserves live cell and dead cell numbers in lung suspension, but increases the CD4:CD8 ratio in allografts from CD4 + FoxP3+ treg-sufficient hosts

**Figure 1:**
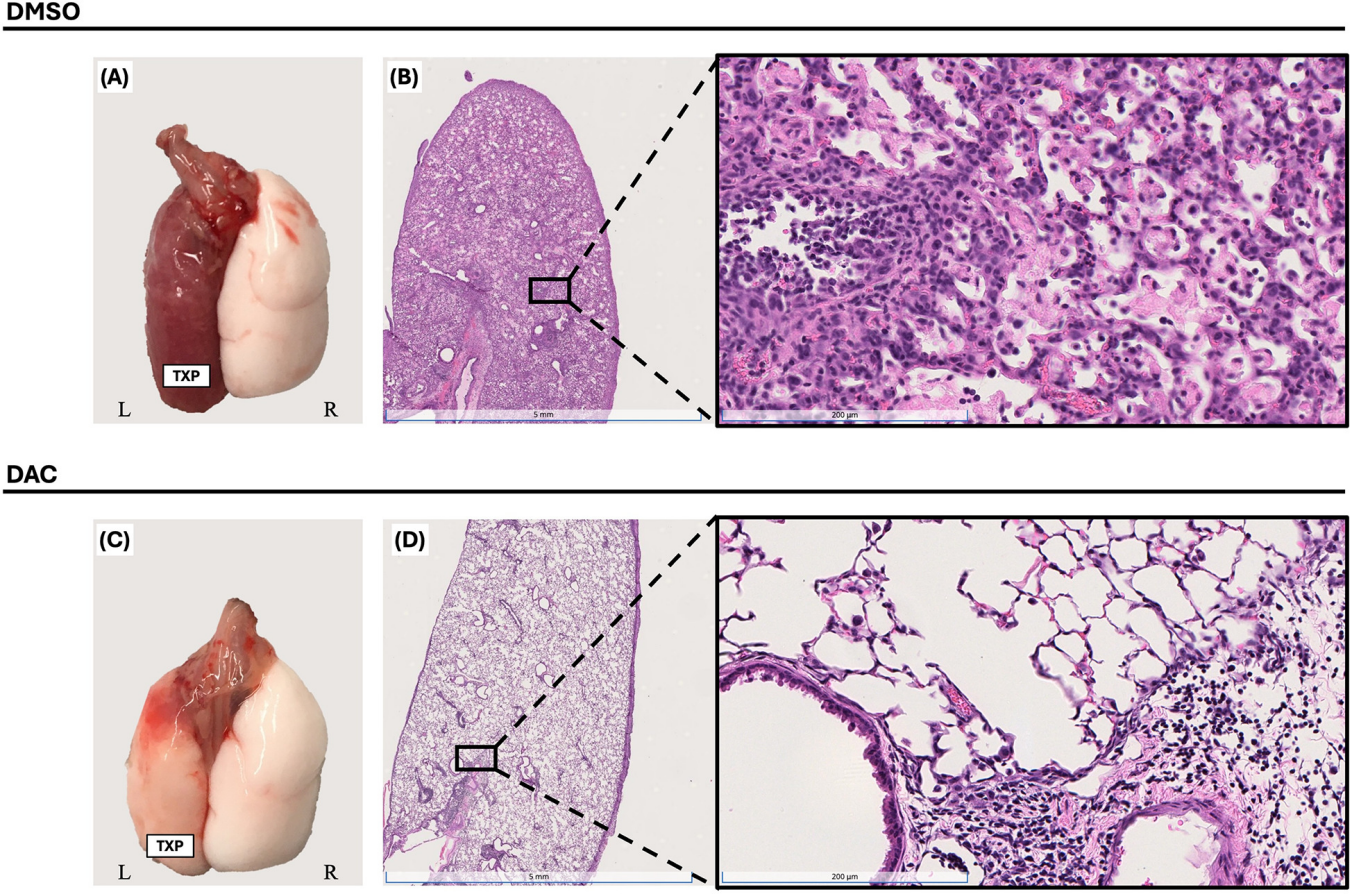
Decitabine attenuates lung allograft rejection. Gross morphology [**(A)** & **(C)**] and histologic hematoxylin and eosin (H&E) staining [**(B)** & **(D)**, 1× and 20× magnifications] of BALB/c lung allografts harvested 10 days post-transplantation into wild-type C57BL/6 hosts. Mice were treated with either vehicle (DMSO, intraperitoneally) or decitabine (DAC, 1 mg/kg, intraperitoneally) on post-transplant days 3, 4, 5, and 8. DAC treatment preserved lung architecture and reduced inflammatory cell infiltration compared to DMSO-treated controls. *TPX: Left Lung Allograft, L: Left, and R: Right*.

This lung transplant model leads to allograft failure with gross lung consolidation (Figures 1A,C) and diffuse dense cellular infiltration histologically (Figures 1B,D). DAC treatment, initiated on post-operative day 3 (POD3), significantly attenuated allograft injury observed 10 days post-transplantation in CD4 + FoxP3+ Treg-sufficient.

**Figure 2:**
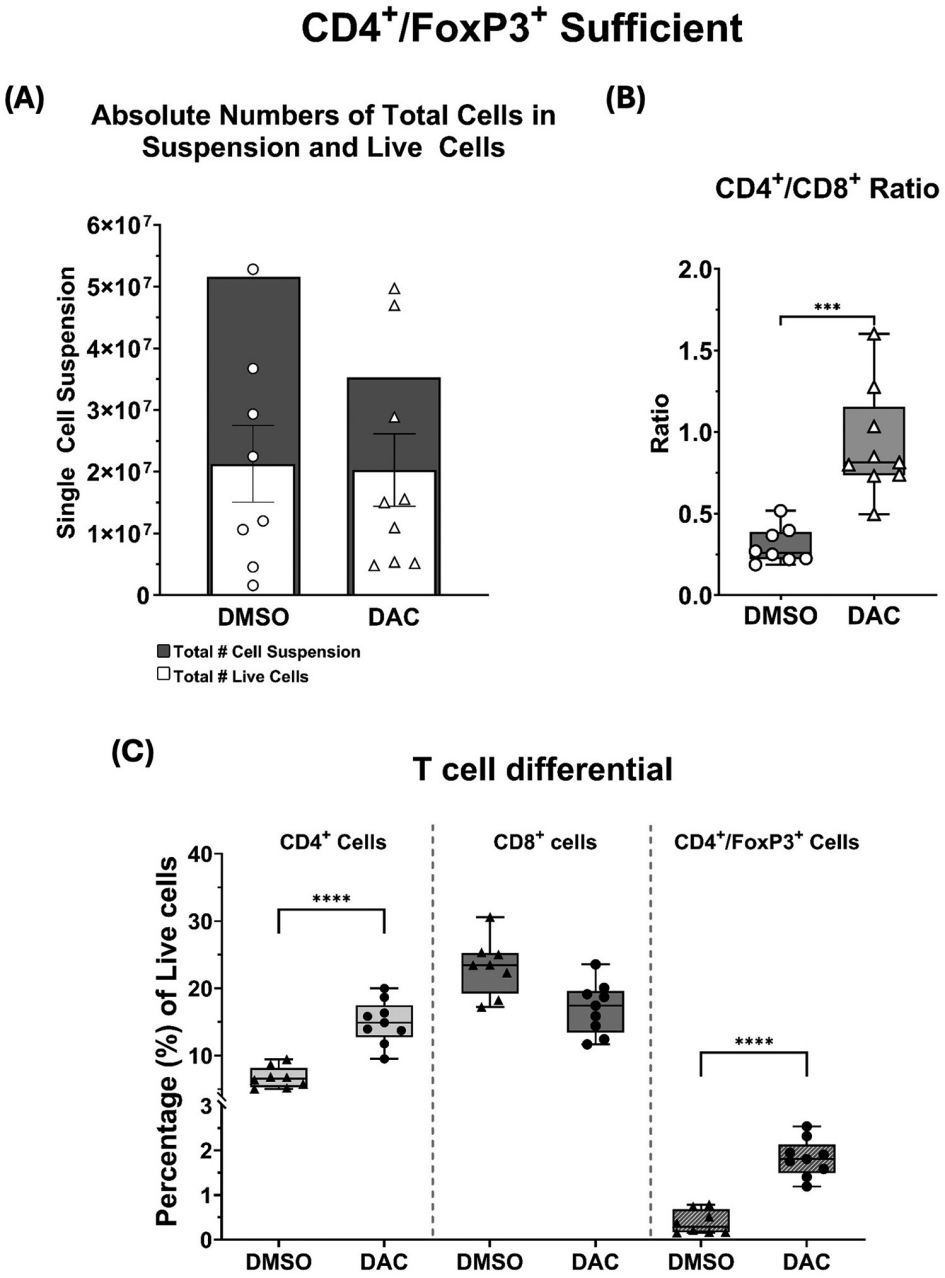
**(A)** Histograms depicting the total single-cell suspension counts (gray histogram) and absolute number of live cells (white histogram). **(B)** Box and whiskers showing CD4^+^/CD8^+^ ratios, and **(C)** the percentages of live CD4^+^, CD8^+^, and CD4^+^FoxP3^+^ T cells as a function of host treatment regimen. *Data are presented as mean* *±* *SEM (N* *=* *6–9 per group). P* ≤ 0.05 (*), P ≤ 0.01 (**), P ≤ 0.001 (***), and P ≤ 0.0001 (****); ns *=* *not significant.*

To explore the effect of host DAC therapy on allograft CD4 + and CD8+ T cell viability, single-cell suspensions from allografts were analyzed using live/dead staining and surface markers for CD4 and CD8. Compared to allografts from DMSO-treated hosts, those from DAC-treated hosts had similar numbers of total dead cells (5.16 ± 0.06 × 10^7^ vs. 3.53 ± 0.7 × 10^7^, *p* = 3.15) and live cells(2.13 ± 0.2 × 10^7^ vs. 2 ± 0.6 × 10^7^, *p* = 0.22) (Figure 2A). Additionally, compared to allografts from DMSO-treated hosts, DAC therapy increased the allograft CD4:CD8T cell ratio (0.30 ± 0.04 vs. 0.93 ± 0.11, *P* = 0.0002) (Figure 2B) predominately by increasing the percentage of live cells that are CD4+ T cells (6.7 ± 0.55% vs. 15.6 ± 0.95%, *P* = 0.0103) (Figure 2C).

Furthermore, DAC treatment significantly increased the percentage of CD4 + FoxP3+ T cells in Treg-sufficient hosts (0.39 ± 0.09% vs. 1.83 ± 0.14%, *P* = 0.01) (Figure 2C). Thus, DAC treatment of hosts preserves allograft live cell numbers, reduces allograft dead cell numbers, and increases the percentage of allograft live cells that are CD4 + .

### 3.2 DAC treatment restricts inflammatory T cells to the perivascular region and reduces airway inflammation

**Figure 3:**
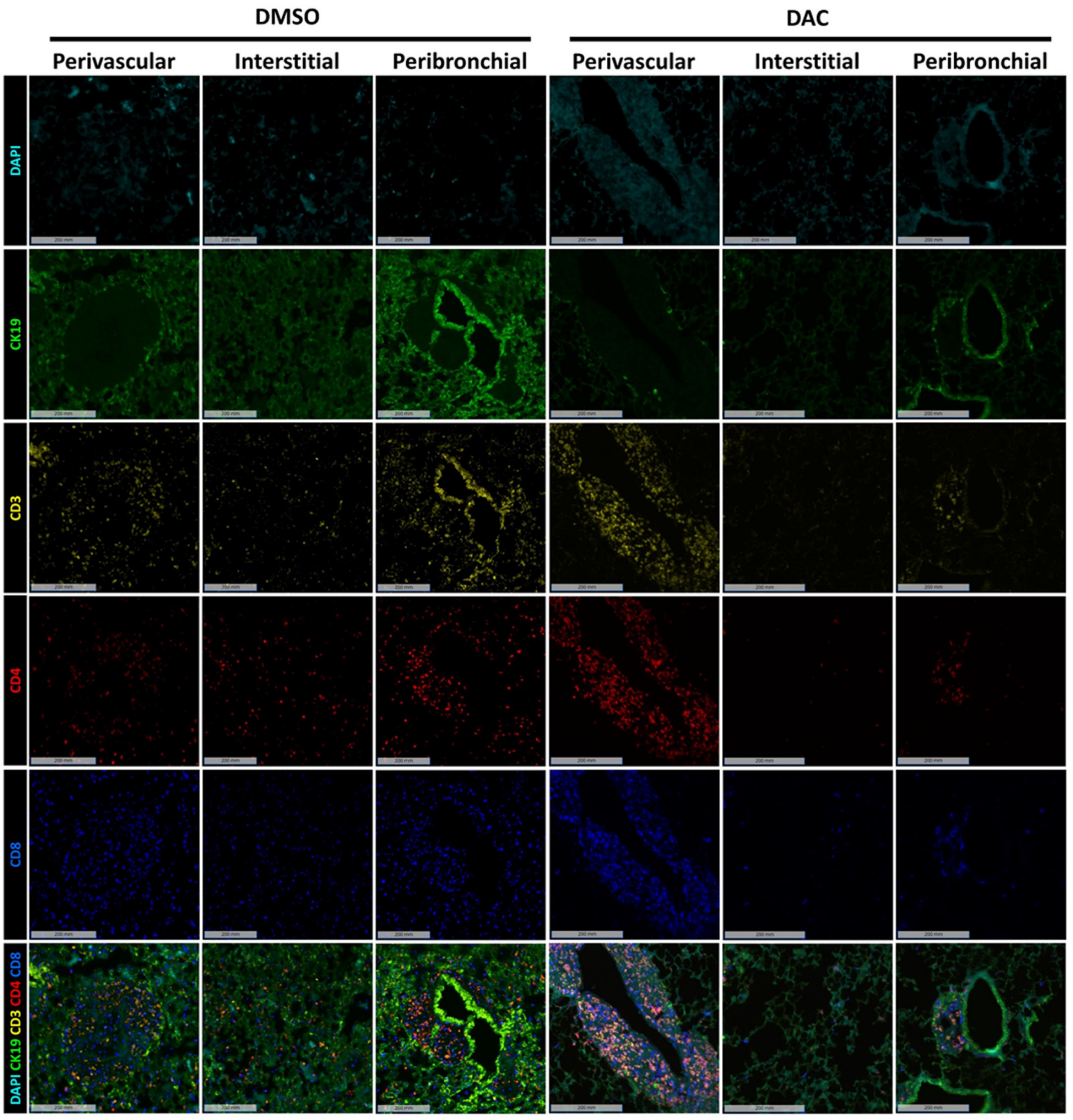
DAC treatment restricts T-cell infiltration to the perivascular region and decreases airway inflammation. Representative immunofluorescence images of allografts harvested 10 days post-transplant from DMSO- and DAC-treated CD4^+^FoxP3^+^ Treg-sufficient hosts. Sections were stained for CK-19 (green), CD3 (yellow), CD4 (red), and CD8 (blue) to **assess** T-cell distribution across different lung regions. DAPI (cyan) marks nuclear staining. *Scale bars* *=* *200 um*.

**Figure 4:**
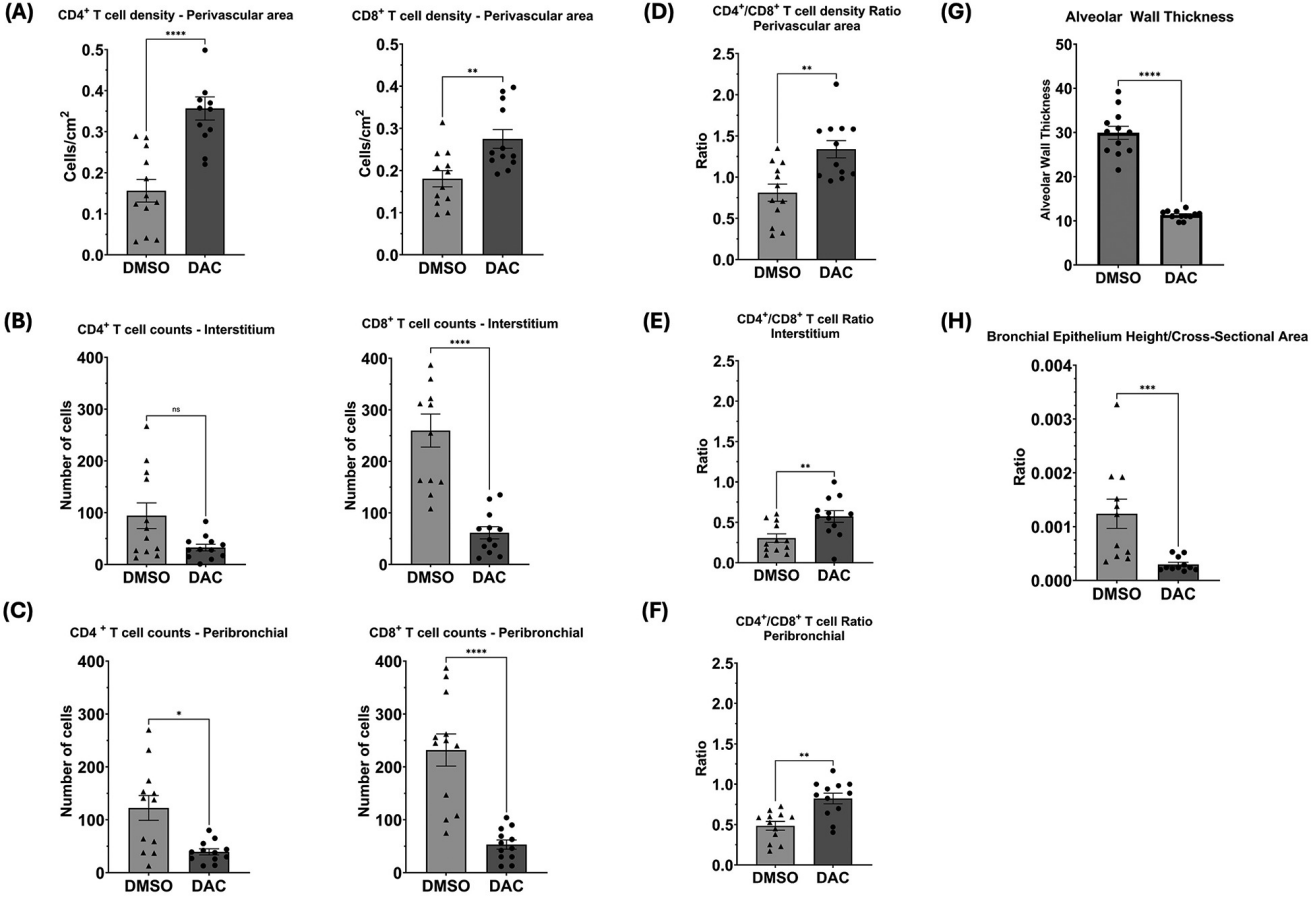
Histograms quantifying the effect of host DAC- (vs. DMSO-) treatment on T cell distribution and airway thickness in allografts. Histograms depict cell counts in **(A)** perivascular, **(B)** interstitial, and **(C)** peribronchial regions, as well as CD4:CD8 ratios **(D, E, & F)**, and **(G)** alveolar wall thickness and **(H)** bronchial epithelial height/cross-sectional area. Four random sites per slide were analyzed, each covering an average total area of 2,400 cm^2^. Perivascular T cell density was measured as CD4^+^ and CD8^+^ T cells per cm^2^ of perivascular area using ImageJ's manual cell count plugin at 20× magnification, with counts normalized to the perivascular area measured, which varied between vessels. Interstitial and peribronchial T cell counts were obtained at 20× magnification and averaged across sites. Alveolar wall thickness was measured in micrometers (µm) at 40× magnification using an Inter-edge Distance Measurement Macro in ImageJ, averaging at least 10 distances per alveolar wall, with five alveoli measured per site. Bronchial epithelial height was also measured in µm at 40× magnification using the same macro, averaging at least 10 distances per bronchial wall, while bronchial cross-sectional area was manually outlined and measured using ImageJ, with values averaged across sites*. Data are presented as mean* *±* *SEM. P* ≤ 0.05 (*), P ≤ 0.01 (**), P ≤ 0.001 (***), and P ≤ 0.0001 (****); ns *=* *not significant*.

Immunofluorescence analysis for T cells markers (CD3, CD4, and CD8) and the epithelial marker CK-19 was performed (Figure 3) showing widespread distribution of T cells throughout all regions of the lung allografts in DMSO-treated hosts. Though most prominent in the perivascular region, T cells were also observed in high density surrounding large airways, dispersed within the interstitium, and infiltrating the alveoli, where CD8+ T cells comprised most intra-alveolar cells. By contrast, in allografts from DAC-treated hosts, T cells were primarily confined to the perivascular region (Figure 4A), with minimal infiltration into the interstitium (Figure 4B), airways, and alveoli (Figure 4C). Additionally, the CD4:CD8 ratio was also significantly increased in the DAC-treated hosts' perivascular (1.33 ± 0.1 vs. 0.81 ± 0.1, *P* = 0.0056) (Figure 4D), interstitial (0.57 ± 0.07 vs. 0.3 ± 0.05, *P* = 0.0083) (Figure 4E), and peribronchial (0.82 ± 0.07 vs. 0.49 ± 0.05, *P* = 0.0011) (Figure 4F) regions compared to DMSO-treated mice.

Immunostaining for CK-19 revealed that the airways (alveolar and bronchial) of allografts from DMSO-treated hosts appeared thickened compared to those from the DAC-treated mice. This effect was evidenced by increased thickness of type I and type II alveolar epithelial cells (alveolar wall thickness) (Figure 4G) and in the bronchiolar epithelial cell height (epithelial cell length: bronchiole area ratio) (Figure 4H).

### 3.3 DAC-treatment modulates host T cell cytokine responses

**Figure 5:**
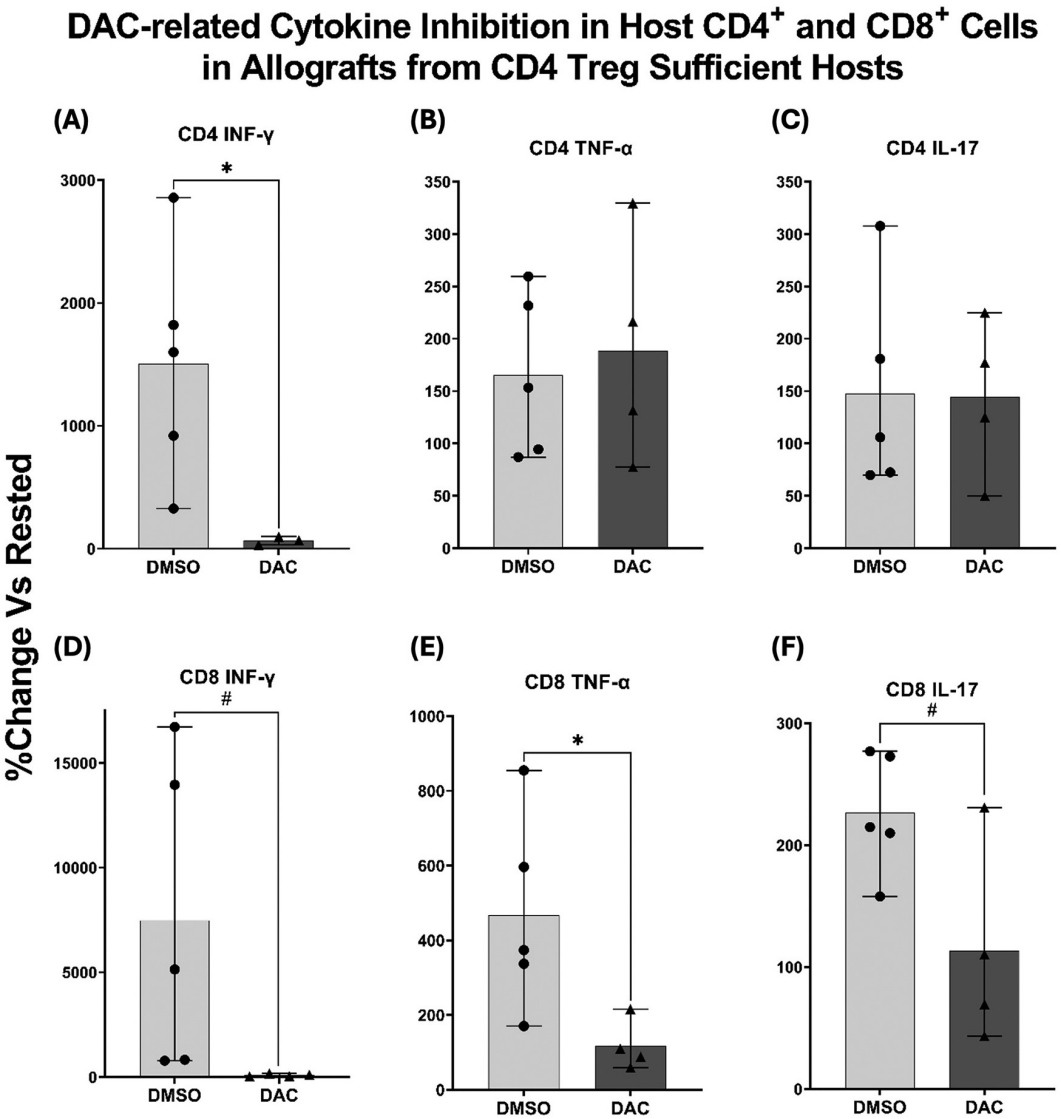
DAC treatment inhibits cytokine production in host CD4^+^ and CD8^+^ T cells. Histograms displaying percentage change in cytokine production from rested CD4^+^ FoxP3^+^ Treg-sufficient host right lung cells after exposure to BALB/c splenocytes. Production of IFN-*γ*, TNF-α, and IL-17 was assessed in **(A–C)** CD4^+^ T cells and **(D–F)** CD8^+^ T cells. Native right lungs from host wild-type C57BL/6, having received BALB/c orthotopic left lung transplants and being treated with DMSO or DAC, were harvested. Their cells were incubated overnight (in R10 medium) and cultured with BALB/c spleen cells (1:1 ratio, 37°C, 5% CO2 × 5 h with GogliStop^TM^ present the final 2 h). Cells were then harvested, stained for surface antibodies and intracellular cytokines, and analyzed with flow cytometry. *Data are presented as mean ± SEM, with individual data points overlaid. P *≤ 0.05 (*), 0.05 < P < 0.09 (#). N = 4–5 per group.

Both CD4 + and CD8 + responder T cells from the native lungs of DMSO-treated hosts demonstrated INF-*γ*, TNF-α, and IL-17 production when incubated with BALB/c splenic stimulator cells. When responder T cells from the native lungs were obtained from DAC-treated hosts: (1) INF-*γ* production was statistically suppressed in CD4+ T cells (Figure 5A) and trended towards suppression in CD8+ T cells (*P* < 0.09) (Figure 5D); (2) TNF-α persisted in CD4+ T cells (Figure 5B) but was markedly suppressed in CD8+ T cells (Figure 5E; (3) IL-17 production persisted in CD4+ T cells (Figure 5C) but trended towards being suppressed in CD8+ T cells (*P* < 0.065) (Figure 5F). Overall, DAC treatment suppressed T cell responses more broadly in allograft CD8+ T cells than in CD4+ T cells.

### 3.4 DAC treatment requires host Cd4 + FoxP3+ treg-sufficiency to maximally abrogate lung allograft rejection

**Figure 6:**
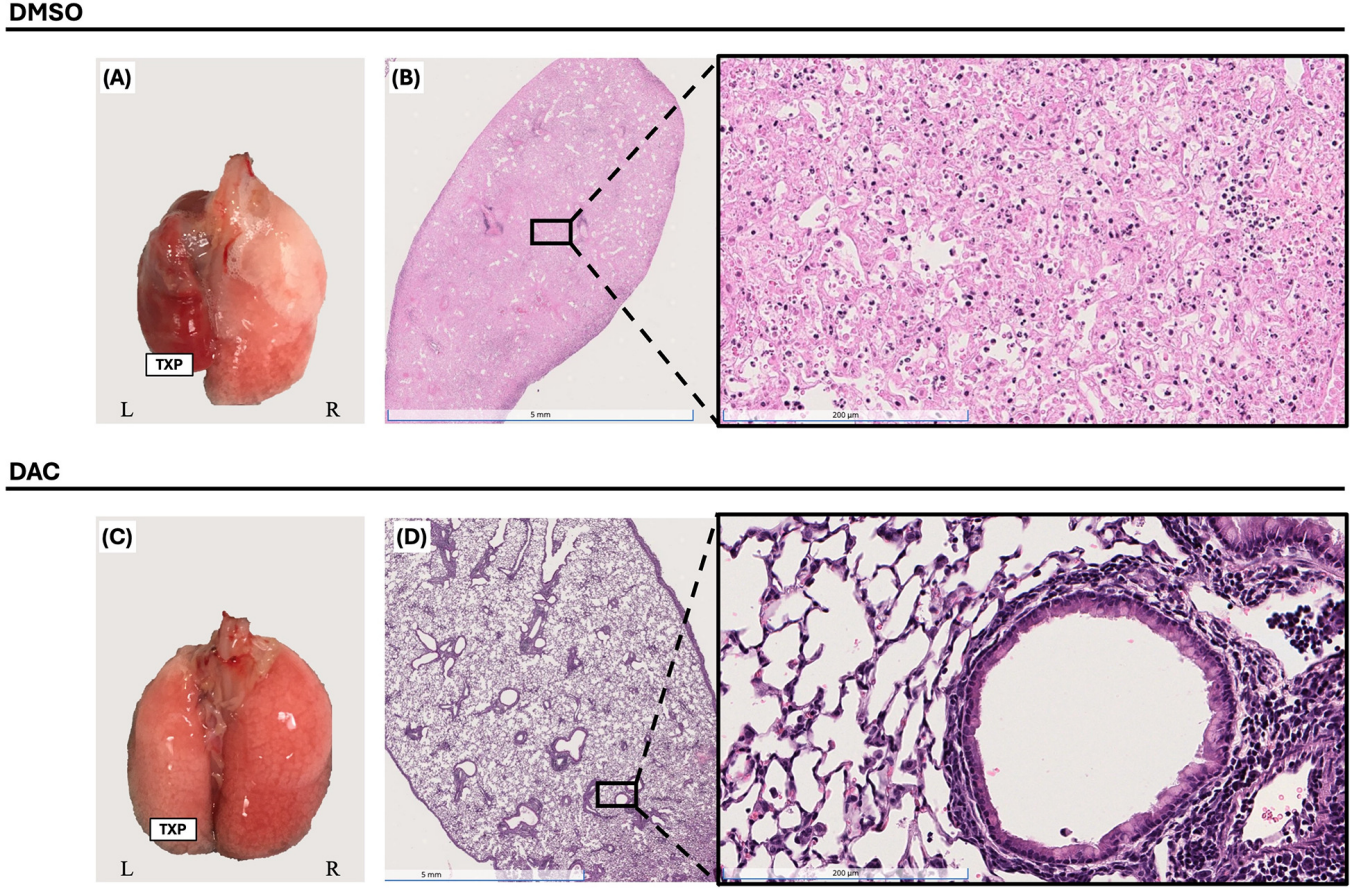
Decitabine inhibition of lung allograft rejection requires CD4 ^+^ FoxP3^+^ treg-sufficient host for maximal effect. Gross morphology [**(A)** & **(C)**] and histologic hematoxylin and eosin (H&E) staining [**(B)** & **(D)**, 1× and 20× magnifications] of BALB/c lung allografts harvested 10 days post-transplantation from *FoxP3^DTR^* hosts. Mice were treated with either DMSO (vehicle, intraperitoneally) or decitabine (DAC, 1 mg/kg, intraperitoneally) on post-transplant days 3, 4, 5, and 8. Hosts also received diphtheria toxin (dT) on post-transplant days 3 (20 ng/kg), 5 (10 ng/kg), and 7 (10 ng/kg) for CD4^+^FoxP3^+^ Treg depletion. *TPX: Left Lung Allograft, L: Left, and R: Right.*

**Figure 7:**
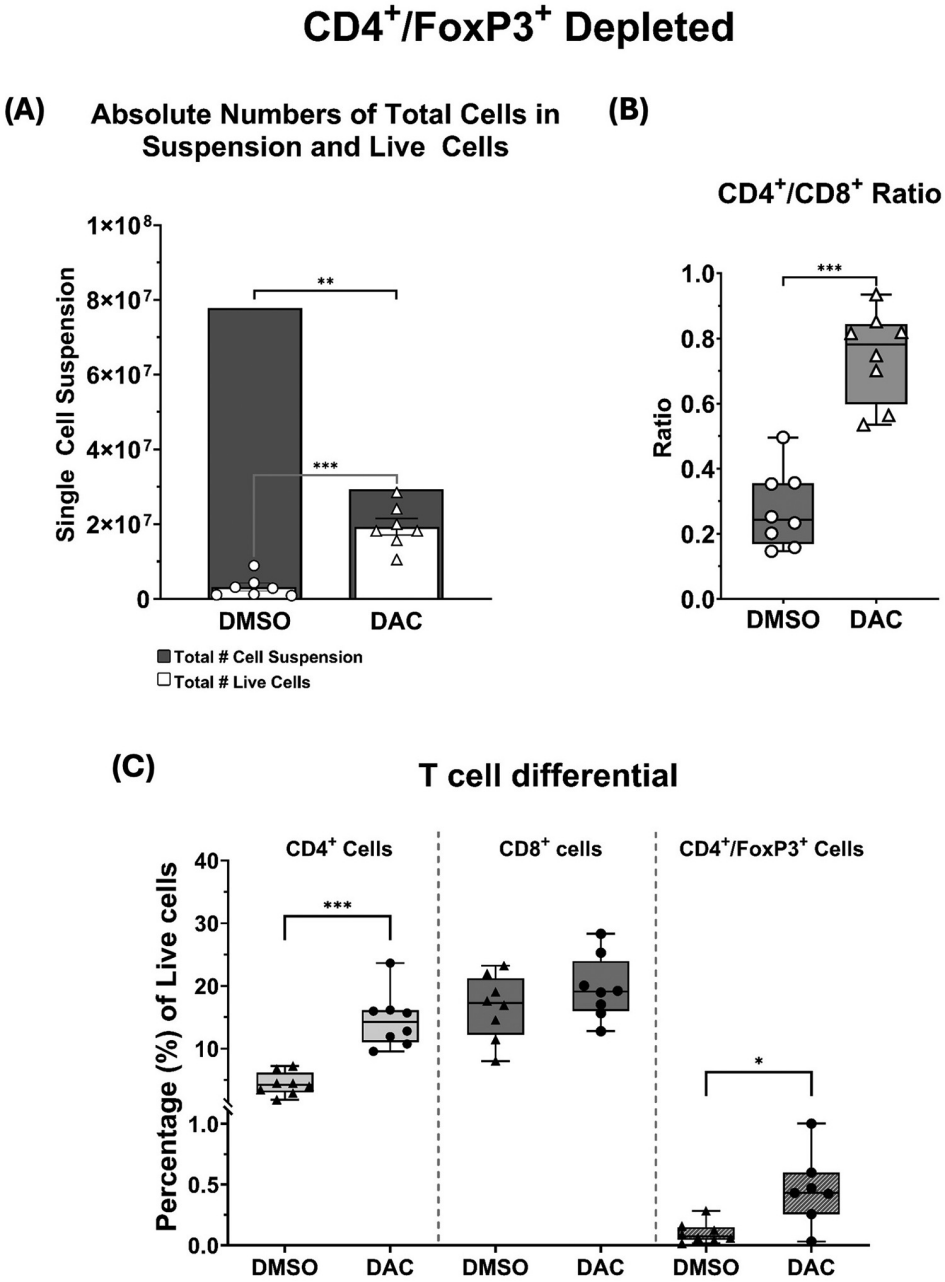
**(A)** Histograms depicting the total single-cell suspension counts (gray histogram) and absolute number of live cells (white histogram). **(B)** Box and whiskers showing CD4^+^/CD8^+^ ratios, and **(C)** the percentages total live cell population composed by CD4^+^, CD8^+^, and CD4^+^FoxP3^+^ T cells. CD4^+^FoxP3^+^ depletion with dT is very effective at removing this cell population*. Data are presented as mean* *±* *SEM (N* *=* *6–9 per group). P* ≤ 0.05 (*), P ≤ 0.01 (**), P ≤ 0.001 (***), and P ≤ 0.0001 (****); ns *=* *not significant*.

FoxP3*^DTR^* host treatment with dT effectively depleted CD4 ^+^ FoxP3^+^ Treg cells in allografts from DMSO treated hosts (Supplementary S3A) and prevented the increase in CD4 + FoxP3^+^ Treg cells typically observed with DAC administration FoxP3*^DTR^* hosts (Supplementary S3B). Host CD4 + FoxP3+ Treg depletion reduced but did not abolish the allograft-protective effect of DAC treatment compared to DMSO as evidence grossly (Figures 6A,C) and histologically (Figures 6B,D) (Supplementary S4A). The effect of DAC treatment on allograft's cell death was most dramatic in allografts from CD4 + FoxP3+ Treg-depleted hosts, where DAC treatment resulted in a 600% increase in live cells (3.2 ± 1.06 × 106 vs. 19.3 ± 2.19 × 106 cells, *P* < 0.009) (Figure 7A). In DMSO-treated hosts, CD4 + FoxP3+ Treg depletion was associated with the near-complete loss of living cells in the allograft by post-transplant day 10. In a pattern similar to that seen in allografts of CD4 + FoxP3+ Treg-sufficient hosts, CD4 + FoxP3+ Treg-depleted hosts treated with DAC (compared to DMSO) demonstrated increased CD4:CD8T-cell ratio (0.27 ± 0.04 vs. 0.75 ± 0.05, *P* = 0.0002) (Figure 7B) predominately by increasing the percentage of live cells that are CD4+ T cells (4.3 ± 0.65% vs. 13.3 ± 1.02%, *P* = 0.0026) (Figure 7C). These findings confirm that, while DAC treatment requires host CD4 + FoxP3+ Treg-sufficiency to provide its maximal salutary effects, the initiation of DAC therapy in hosts of acutely rejecting lung allografts reduces cellular lung allograft rejection by mechanisms independent of host CD4 + FoxP3+ T cells. Notably, the dT therapy used to deplete CD4 + FoxP3+ Tregs in Foxp3*^DTR^* mice did not alter allograft histology when administered to C57BL6 wild-type mice (Supplementary S5). Additionally, native lungs in DMSO-treated hosts were similarly injured whether the host was CD4 + FoxP3+ Treg-sufficient or -depleted (Supplementary S4B).

### 3.5 Treatment of CD4 + FoxP3+ treg-sufficient hosts with DAC does not greatly change marker expression on live allograft CD4 + FoxP3 + or live allograft CD8 + FoxP3 + cells

**Figure 8:**
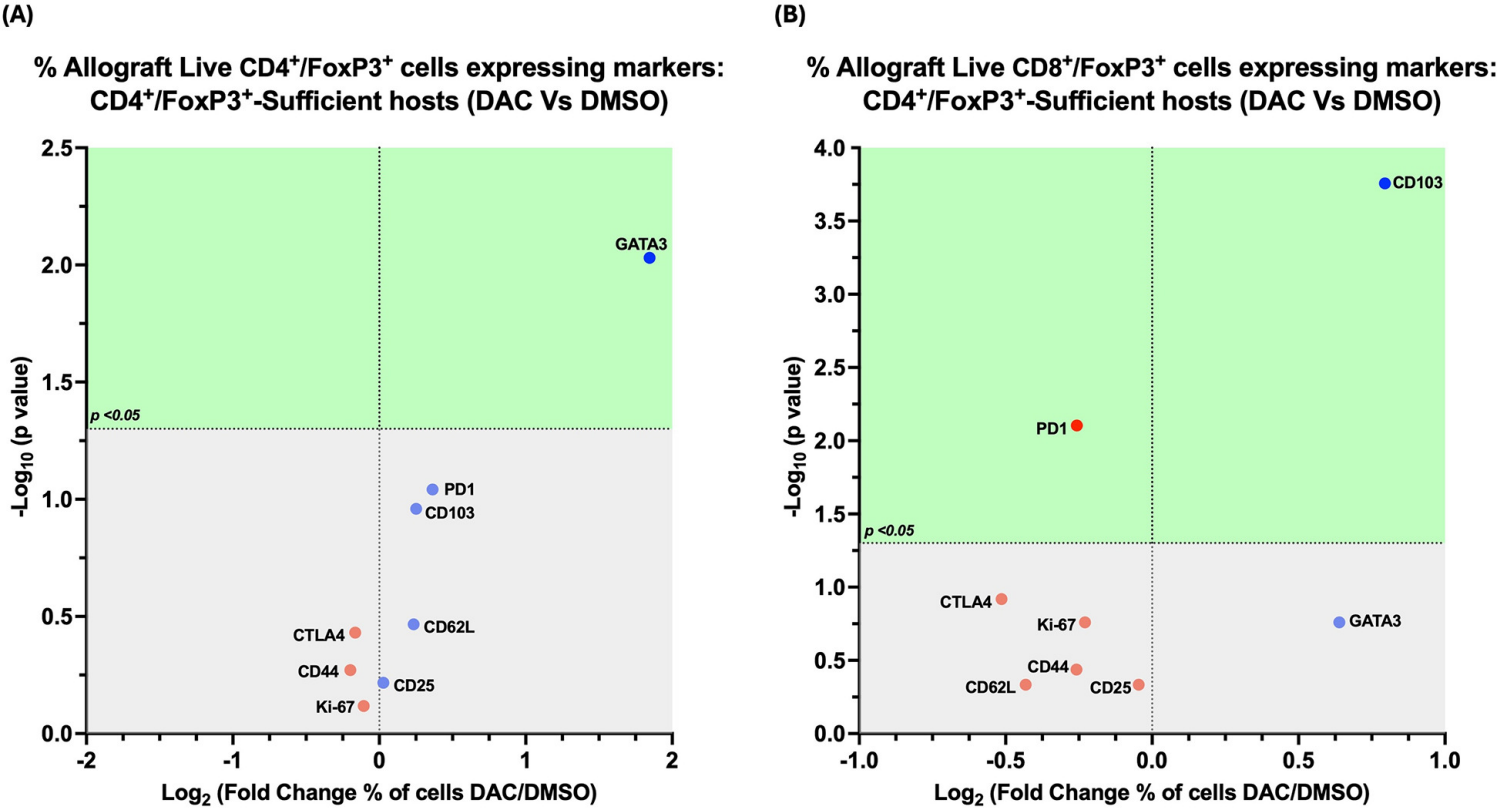
Effect of DAC on the phenotype of CD4 ^+^ FoxP3^+^ T cells in CD4 ^+^ FoxP3^+^ treg sufficient hosts. Volcano plot demonstrating the effect on the percentage of allograft live CD4^+^FoxP3^+^ T cells **(A)** and on live CD8^+^FoxP3^+^ T cells **(B)** of DAC vs. DMSO treatment of CD4^+^FoxP3^+^ Treg-sufficient hosts. Effect (*x*-axis) is presented as Fold Change (Log_2_) in the percentage of live allograft cells expressing a given marker when the host is treated with DAC rather than DMSO. Markers expressed on a higher percentage of cells are depicted more to the right, and increasing statistical significance is depicted by ascending position on the *Y* axis. The green-shaded region denotes markers with statistically significant differences (*p* < 0.05, -log_10_ > 1.301). Markers with increased expression in DAC-treated hosts are shown in blue, while those with reduced expression are in red.

The percentage of allograft FoxP3 + cells from DAC- vs. DMSO- treated hosts expressing the immune markers we queried was minimally changed by DAC treatment. Host treatment with DAC increased only the percentage of CD4 + FoxP3+ T cells expressing GATA3 (Figure 8A), the percentage of CD8 + FoxP3+ T cells expressing CD103, and the percentage of CD8 + FoxP3+ T cells not expressing PD1 (Figure 8B).

### 3.6 Host's depletion of CD4 + FoxP3+ T cells modifies the effect of host's DAC treatment on allograft live CD4+ T cells expressing various markers

**Figure 9:**
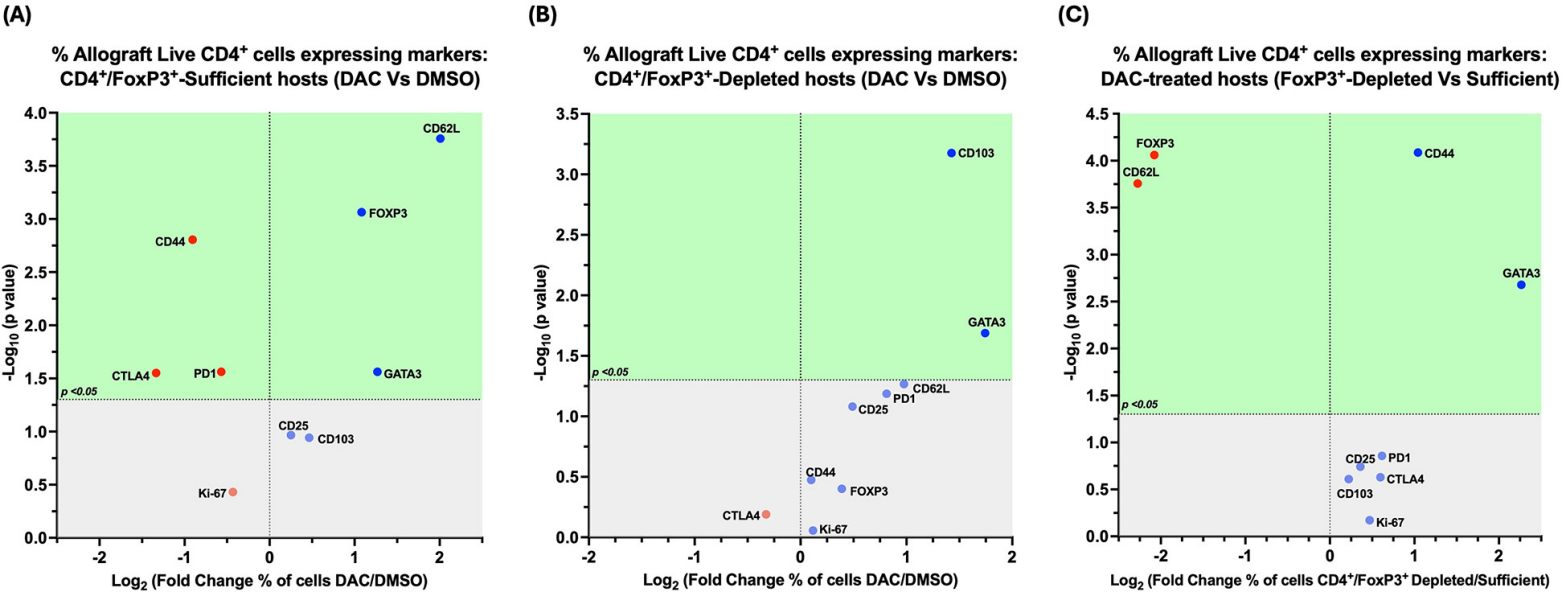
Cd4 ^+^ FoxP3^+^ treg depletion alters the effect of DAC treatment on allograft CD4^+^ T cells. Volcano plot demonstrating the effect on percentage of allograft live CD4^+^ T cells expressing various markers following **(A)** *DAC (*vs. *DMSO) treatment of* CD4^+^FoxP3^+^ Treg*-sufficient hosts*; **(B)** *DAC (*vs. *DMSO) treatment of* CD4^+^FoxP3^+^ Treg*-depleted hosts;* **(C)** *DAC treatment of* CD4^+^FoxP3^+^ Teg*-sufficient* vs. *–depleted hosts*. Effect (*x*-axis) is presented as Fold Change (Log_2_) in the percentage of live CD4^+^ allograft cells expressing a given marker when the **(A)** CD4^+^FoxP3^+^ Treg–sufficient host is treated with DAC (vs. DMSO); **(B)** CD4^+^FoxP3^+^ Treg-depleted host is treated with DAC (vs. DMSO); **(C)** CD4^+^FoxP3^+^ Treg-sufficient vs. –depleted host is treated with DAC. Markers expressed on a higher percentage of cells are depicted more to the right, and increasing statistical significance is depicted by ascending position on the *Y* axis (-Log_10_). The green-shaded region denotes markers with statistically significant differences (*p* < 0.05, -log_10_ > 1.301). Markers with increased expression in DAC-treated hosts are shown in blue, while those with reduced expression are in red.

In CD4 + FoxP3+ Treg-sufficient hosts, DAC- (vs. DMSO-) treatment resulted in a greater percentage of live allograft CD4+ T cells expressing CD62l, GATA-3, and FoxP3, with a lower percentage expressing CD44, PD1, and/or CTLA-4 (Figure 9A).

In CD4 + FoxP3+ Treg-depleted hosts, DAC- (vs. DMSO-) treatment resulted in a greater percentage of live allograft CD4+ T cells expressing CD103, GATA-3, and/or CD62l (*P* = 0.054) (Figure 9B).

In DAC-treated hosts, the loss of CD4 + FoxP3+ Treg-sufficiency results in a greater percentage of live allograft CD4+ T cells expressing CD44 and/or GATA-3, but a lower percentage expressing CD62l or FoxP3 (Figure 9C).

### 3.7 Host's depletion of CD4 + FoxP3+ T cells modifies the effect of host's DAC treatment on allograft live CD8+ T cells expressing various markers

**Figure 10:**
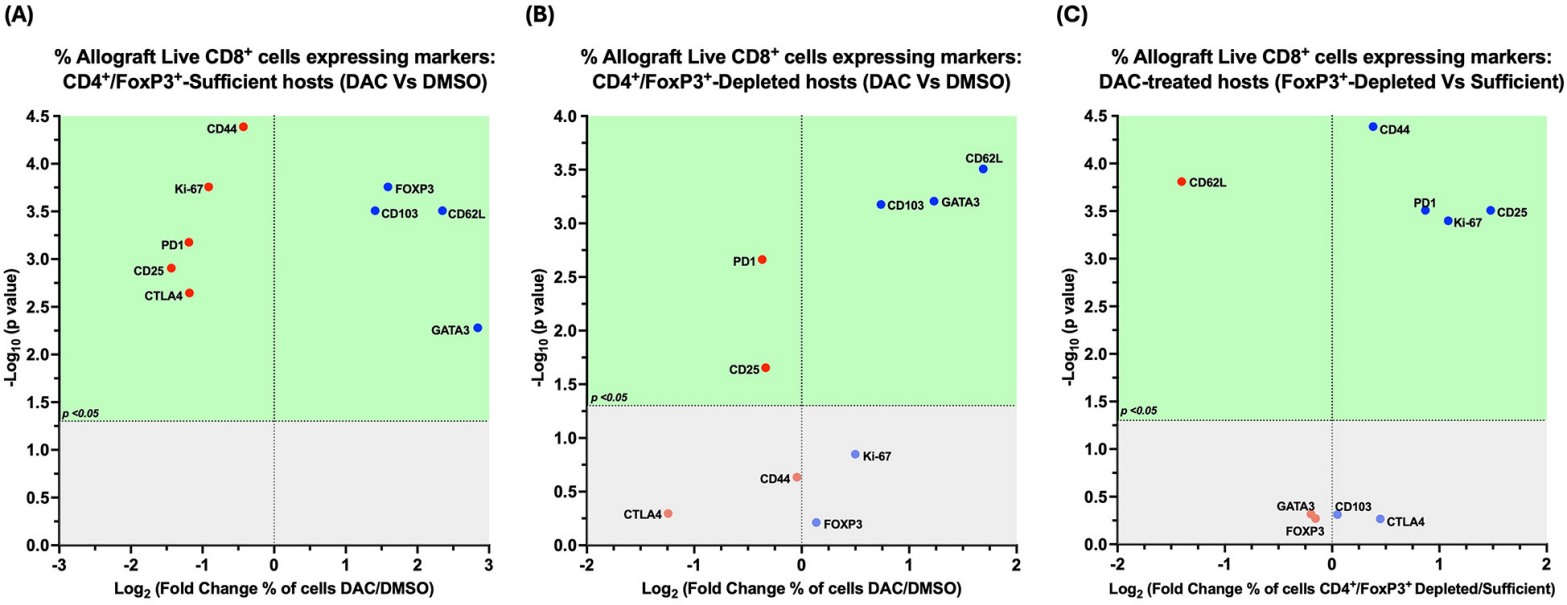
Cd4 ^+^ FoxP3^+^ treg depletion alters the effect of DAC treatment on allograft CD8^+^ T cells. Volcano plot demonstrating the effect on the percentage of allograft live CD8^+^ T cells expressing various markers following **(A)** *DAC (*vs. *DMSO) treatment of* CD4^+^FoxP3^+^ Treg*-sufficient hosts*; **(B)** *DAC (*vs. *DMSO) treatment of* CD4^+^FoxP3^+^ Treg*-depleted hosts*; **(C)** *DAC treatment of* CD4^+^FoxP3^+^ Treg*-sufficient* vs. *–depleted hosts.* Effect (*x*-axis) is presented as Fold Change (Log_2_) in the percentage of live CD8^+^FoxP3^+^ allograft cells expressing a given marker when the **(A)** CD4^+^FoxP3^+^ Treg–sufficient host is treated with DAC (vs. DMSO); **(B)** CD4^+^FoxP3^+^ Treg-depleted host is treated with DAC (vs. DMSO); **(C)** CD4^+^FoxP3^+^ Treg-sufficient vs. –depleted host is treated with DAC. Markers expressed on a higher percentage of cells are depicted more to the right, and increasing statistical significance is depicted by ascending position on the *Y* axis (-Log_10_). The green-shaded region denotes markers with statistically significant differences (*p* < 0.05, -log_10_ > 1.301). Markers with increased expression in DAC-treated hosts are shown in blue, while those with reduced expression are in red.

In CD4 + FoxP3+ Treg-sufficient hosts, DAC- (vs. DMSO-) treatment resulted in a greater percentage of live allograft CD8+ T cells expressing CD103, CD62l, GATA-3, and FoxP3, with a lower percentage expressing Ki-67, CD25, CD44, PD1, and/or CTLA-4 (Figure 10A).

In CD4 + FoxP3+ Treg-depleted hosts, DAC- (vs. DMSO-) treatment resulted in a greater percentage of live allograft CD8+ T cells expressing CD103, GATA-3, and/or CD62l, with a lower percentage expressing CD25 and/or PD1 (Figure 10B).

In DAC-treated hosts, the loss of CD4 + FoxP3+ Treg-sufficiency results in a greater percentage of live allograft CD8+ T cells expressing CD44, CD28, Ki-67, and PD1, but a lower percentage expressing CD62l and FoxP3 (Figure 10C).

### 3.8 Host’ depletion of CD4 + FoxP3+ T cells modifies the effect of host's DAC treatment on allograft live CD8 + FoxP3+ T cells expressing various markers

**Figure 11:**
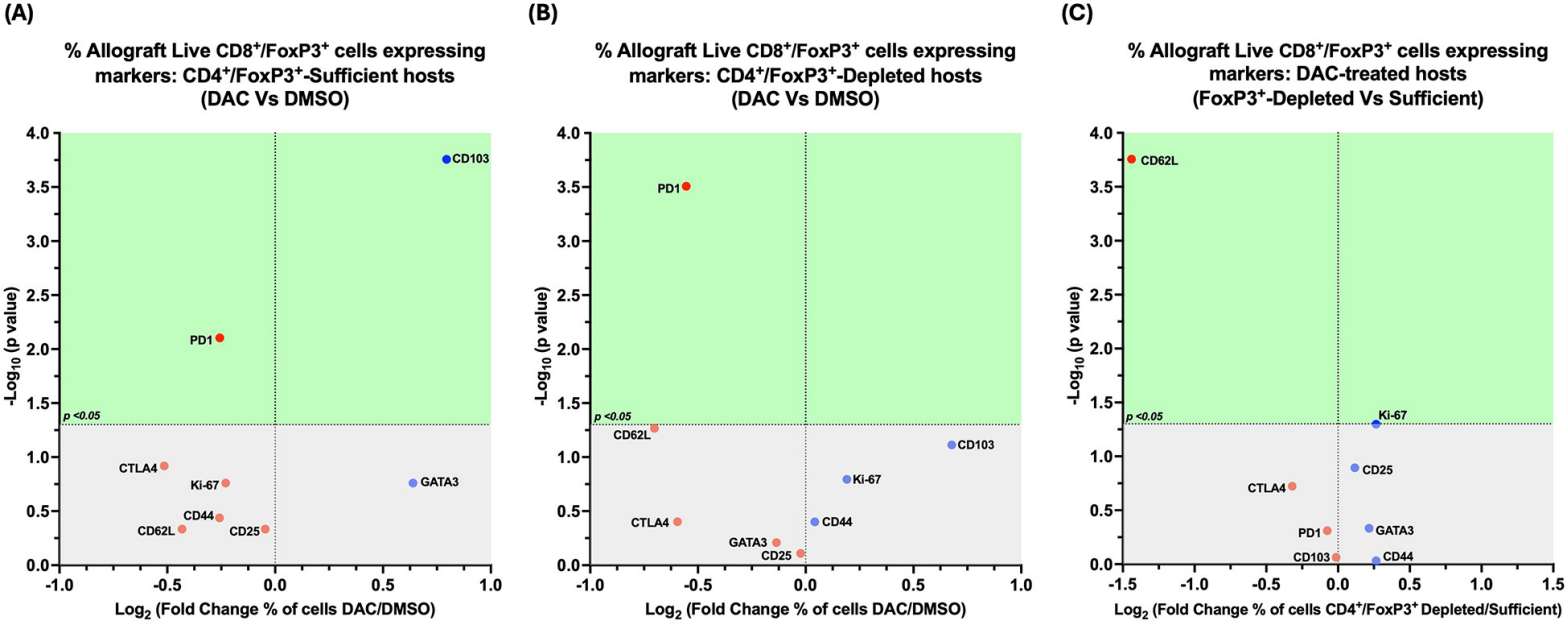
Cd4 ^+^ FoxP3^+^ treg depletion alters the effect of DAC treatment on allograft's CD8^+^FoxP3^+^ T cells. Volcano plot demonstrating the effect on the percentage of allograft live CD8^+^FoxP3^+^ T cells expressing various markers following **(A)** *DAC (*vs. *DMSO) treatment of* CD4^+^FoxP3^+^ Treg*-sufficient hosts*; **(B)** *DAC (*vs. *DMSO) treatment of* CD4^+^FoxP3^+^ Treg*-depleted hosts*; **(C)** *DAC treatment of* CD4^+^FoxP3^+^ Treg*-sufficient* vs. *–depleted hosts.* Effect (*x*-axis) is presented as Fold Change (Log_2_) in the percentage of live CD8^+^FoxP3^+^ allograft cells expressing a given marker when the **(A)** CD4^+^FoxP3^+^ Treg–sufficient host is treated with DAC (vs. DMSO); **(B)** CD4^+^FoxP3^+^ Treg-depleted host is treated with DAC (vs. DMSO); **(C)** CD4^+^FoxP3^+^ Treg-sufficient vs. –depleted host is treated with DAC. Markers expressed on a higher percentage of cells are depicted more to the right, and increasing statistical significance is depicted by ascending position on the *Y* axis (-Log_10_). The green-shaded region denotes markers with statistically significant differences (*p* < 0.05, -log_10_ > 1.301). Markers with increased expression in DAC-treated hosts are shown in blue, while those with reduced expression are in red.

In CD4 + FoxP3+ Treg-sufficient hosts, DAC- (vs. DMSO-) treatment resulted in a greater percentage of live allograft CD8 + FoxP3+ T cells expressing CD103, with a lower percentage expressing PD1 (Figure 11A).

In CD4 + FoxP3+ Treg-depleted hosts, DAC- (vs. DMSO-) treatment resulted in a lower percentage of live allograft CD8 + FoxP3+ T cells expressing PD1 (Figure 11B).

In DAC-treated hosts, the loss of CD4 + FoxP3+ Treg-sufficiency results in a lower percentage of live allograft CD8 + FoxP3+ T cells expressing CD62l (Figure 11C).

### 3.9 DAC-treatment of hosts shifts allograft CD8+ T cells toward an anti-inflammatory phenotype

**Figure 12:**
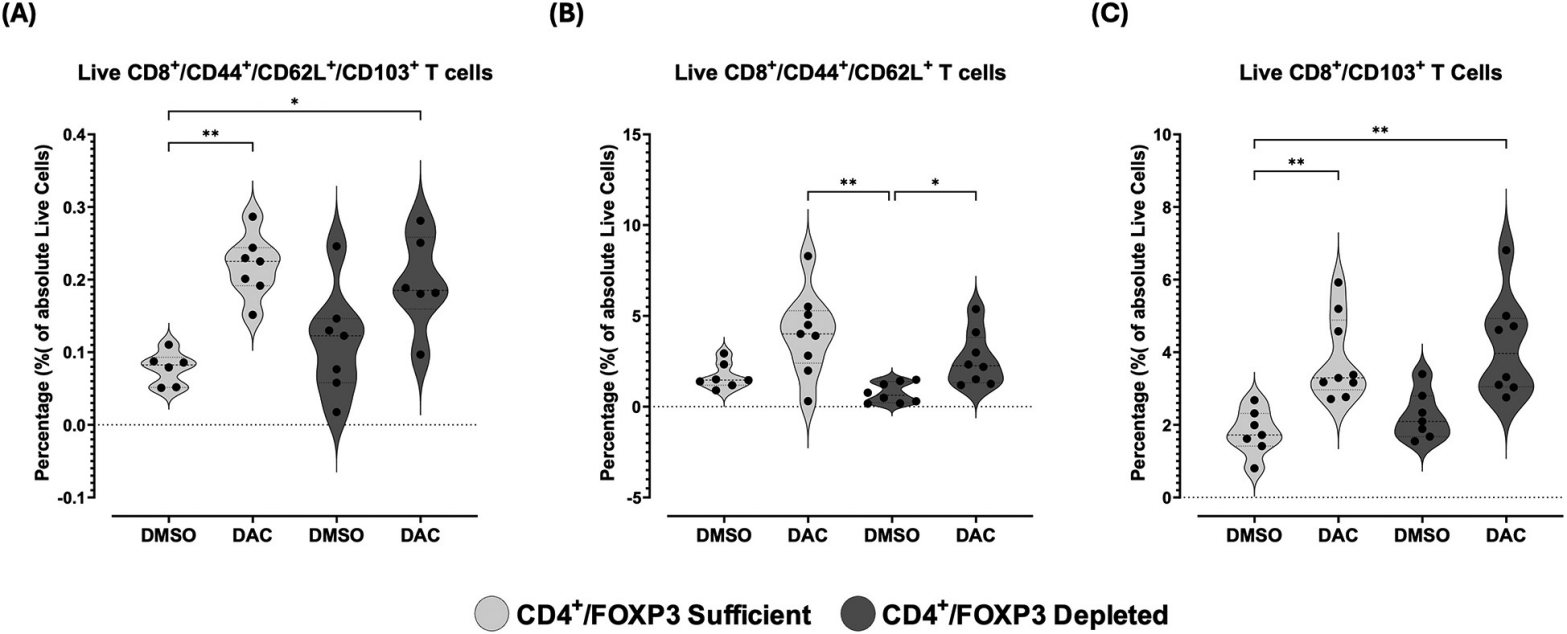
DAC treatment increases the percentage of CD8^+^ T cells expressing markers of immune tolerance in CD4 ^+^ FoxP3^+^ treg-sufficient and -depleted hosts. Violin plots display the percentage of allograft live **(A)** CD8 ^+^ CD44 ^+^ CD62l ^+^ CD103^+^, **(B)** CD8 ^+^ CD44 ^+^ CD62l^+^, and **(C)** CD8 ^+^ CD103^+^ T-cells in transplanted lungs 10 days post-transplant. CD4 ^+^ FoxP3^+^ Treg-sufficient hosts were wild-type C57BL/6 mice treated with either diluent (DMSO) or DAC. CD4 ^+^ FoxP3^+^ Treg-depleted hosts were diphtheria toxin (dT)-treated FoxP3^DTR^ mice receiving either diluent or DAC. DAC (1 mg/kg, i.p.) or DMSO was administered on post-transplant days 3, 4, 5, and 8, while dT was given on post-transplant days 3 (20 ng/kg), 5 (10 ng/kg), and 7 (10 ng/kg). *Data are presented as median, minimum, and maximum with individual data points overlaid. P* ≤ 0.05 (*), P ≤ 0.01 (**), P ≤ 0.001 (***), and P ≤ 0.0001 (****); ns *=* *not significant*.

Having observed an overall trend towards the promotion of anti-inflammatory markers on live CD8+ T cells in allografts of DAC-treated hosts, we queried whether DAC administration to the host affected the relative magnitude of specific combinations of immunosuppressive markers characteristic of immunosuppressive CD8-phenotype populations. DAC treatment of CD4 + FoxP3+ Treg-sufficient hosts significantly increased the percentage of allograft live CD8+ T cells expressing CD44 + CD62l + CD103+ (Figure 12A), while DAC treatment of CD4 + FoxP3+ Treg-deficient hosts significantly increased the percentage of live allograft CD8+ T cells expressing CD44 and CD62l without CD103 (Figure 12B). This suggests that either a direct effect of DAC on CD8+ T cell expression of CD103 + marker is modified by CD4 + FoxP3+ T cells, or that DAC acts through CD4 + FoxP3+ Tregs to regulate CD8+ T cell phenotype (Figure 12C).

## 4 Discussion

We demonstrate that DAC, initiated even 72 h following graft implantation, attenuates murine lung allograft rejection seen at 10 days through a process that requires sufficient host CD4 + FoxP3+ T cells to maximize its effect. The attenuated rejection is accomplished through a process that: (1) leads to a rise in the percentage of live cells that are CD4+ T cells; (2) confines CD4 + and CD8+ T cells to the perivascular rather than to the interstitial lung regions; (3) transitions CD4 + and CD8+ T cells to a less cytotoxic phenotype (which is more dramatic in CD8+ T cells); and (4) requires CD4 + FoxP3+ Tregs for maximal effect.

DAC administration was associated with an increase in CD4+ T cells and an increase in CD4:CD8T cell ratio to nearly 1. An increase in the CD4:CD8T-cell ratio also characterizes the abrogated rejection induced by CD40 costimulatory pathway disruption in this model (15, 16). We find that this increased population of CD4+ T cells possess a less inflammatory cytokine profile, producing less INF-*γ* and more frequently expressing FoxP3. Similarly, the cytokine expression of allograft live CD8+ T cells from DAC-treated hosts is much less inflammatory than that from DMSO-treated hosts, with less INF-*γ*, TNF-α and IL-17. *in vitro* studies indicate DAC induces Foxp3 expression in naïve- and activated-CD4+ T cells by inhibiting methylation of a CpG-rich island within the Foxp3 promoter region (17).

We compared the effect of host DAC vs. DMSO treatment on allograft T cell expression of a wide range of immune tolerance markers, including FoxP3 and GATA-3 (18) (transcription factors for anti-inflammatory Tregs and Th2, respectively), CTLA-4 (19) and PD1 (receptors for the anti-inflammatory ligands CD80/CD86 and PD-L1, respectively); CD25, CD62l (20), CD103 (21) (markers of anti-inflammatory tendency). CD44 was analyzed as a T cell marker of inflammation/rejection in transplantation (22, 23), and Ki-67 as an indicator of proliferation (24). To maximize the effect of DAC, we find that sufficiency of host CD4 + FoxP3+ T cells is required. We have shown in an LPS-induced lung injury model that DAC therapy leads to an increase in the number of lung CD4 + CD25 + FoxP3+ T cells as well as an enhancement of their Foxp3 expression, activation state, and suppressive phenotype (13).

In the current model, host treatment with DAC was consistently associated with an increased percentage of allograft live CD4 + and CD8+ T cells expressing of GATA-3. This persisted independent of host's CD4 + FoxP3+ Treg-sufficiency or -deficiency. GATA-3 is the Th2 transcription factor whose relative expression is inversely associated with acute allograft rejection (25). Host treatment with DAC was also associated with an increased percentage of allograft live CD4 + and CD8+ T cells expressing CD62l. This effect, although still present, was not as great in host cells deficient- (vs. sufficient) in CD4 + FoxP3+ T cells. This suggests that host CD4 + FoxP3+ T cells modify the capacity for DAC treatment of hosts to increase the percentage of live allograft CD4+ T cells expressing CD62l. CD4 + FoxP3+ T-cells are suppressive in nature (26). Foxp3 expression can be induced in naïve CD4+ T cells (27), including in CD4 + CD62l + cells (28). The presence of CD62l on FoxP3 cells identifies a particularly immunosuppressive cell type in settings of experimental autoimmune encephalomyelitis (29) and graft-vs.-host disease following allogeneic bone marrow transplant (30), and may have contributed to the benefit of DAC in allografts of CD4 + FoxP3+ Treg depleted hosts.

The breadth of anti-inflammatory markers expressed, and/or not expressed, on live allograft CD8+ T cells from DAC- (vs. DMSO-) treated hosts is much greater than expressed on live allograft CD4+ T cells. This includes the expression of GATA-3 and FoxP3 (transcription factors for Th2 and Treg cells, respectively). It also includes the expression of CD62l and CD103 (cell surface markers commonly associated with anti-inflammatory phenotype). In our studies, host treatment with DAC increased the percentage of live allograft CD8+ T cells expressing CD62l, CD103, and/or GATA-3 whether the host was CD4 + FoxP3+ Treg-sufficient or -depleted.

We observed host treatment with DAC to be associated with an increase in allograft live CD8 + and CD8 + FoxP3+ T cells expressing CD103 as long as the host is CD4 + FoxP3+ Treg-sufficient. This suggests either a direct effect of DAC on CD8+ T cell expression of CD103 + marker is modified by CD4 + FoxP3+ T cells, or that DAC acts through CD4 + FoxP3+ Tregs to regulate CD8+ T cell phenotype. CD8 + CD103+ T cells are also immunosuppressive (31) and have been noted to be numerous in recipients tolerant of liver transplants (32). Still, allografts from DAC treated hosts continued to show increased percentage of allograft live CD8+ T cells expressing CD44 + CD62l + whether the host is CD4 + FoxP3+ Treg-sufficient or -deficient. The capacity for DAC to reduce CD8+ T cell cytotoxicity may be particularly important in CD4 + FoxP3+ Treg-depleted hosts. Recruitment of CD8 + CD44 + CD62l + T cells into murine lung allografts is a critical early step in the successful development of tolerance in a transplant model using costimulatory blockade (CTLA-4 + MR1) (33). CD8 + CD103+ T cells have themselves been shown to play a role in suppressing the graft-vs.-host disease of a lupus-like syndrome (34, 35) as well as in spontaneous tolerance of liver allografts (32). CD8 + CD103+ T cells don't require the expression of FoxP3 to manifest a suppressive phenotype (36), and CD103 + expression on CD8 + CD103- T cells can be induced by *in vitro* allostimulation (37). The improved histology we observed in allografts of DAC treated hosts was associated with increased expression of CD103 alone, expression of CD44 + CD62+, and expression of CD44 + CD62l + CD103 + on live CD8+ T cells both in allografts from CD4 + FoxP3+ Treg-sufficient and -depleted hosts, although the density of CD103 + expression on live CD8+ T cells is greatest in allografts from CD4 + FoxP3+ Treg-depleted hosts.

In the floxed version of FoxP3 deletion mice we used, the FoxP3 mRNA targeted is present in CD4 + CD25+ T cells and absent in CD8+ T cells (38). Though FoxP3 can be induced in CD8+ T cells by T cell receptor stimulation (39), this is felt to represent emergence from the conventional T cell pool in the periphery (40). The very concept that CD8 + FoxP3+ T cells are universally suppressive has been questioned, as the observed suppression by induced CD8 + FoxP3+ T cells vary from strong (41) to weak (40) in GVHD models (depending on the mode of stimulation). The CD8 + FoxP3+ T cells in our allografts from hosts receiving DAC (CD4 + FoxP3+ Treg-sufficient or -depleted) show enhanced expression of some markers, such as CD103, characteristic of CD8 + FoxP3+ T cells, which promote protection of fully MHC-mismatched skin allografts (42). However, unlike the CD8 + FoxP3+ T cells which promote protection of fully MHC-mismatched skin allografts, the CD8 + FoxP3+ T cells in the lung allografts from our DAC-treated hosts (CD4 + FoxP3+ Treg-sufficient or -depleted) demonstrate neither enhanced CTLA-4 expression nor (in CD4 + FoxP3+ Treg-depleted hosts) the capacity to induce conventional CD4 + FoxP3+ T cells. Additionally, the CD8 + FoxP3+ T cells in our allografts from DAC-treated hosts (CD4 + FoxP3+ Treg-sufficient or -depleted) are less frequently PD-1 + than those not receiving DAC. Interestingly, Takahashi et al. found that PD-1 is critical to the tolerogenic effect of costimulatory blockade in a murine lung transplant model, as host CD8+ T cells lacking of PD-1 expression undergo prolonged interaction with graft antigen presenting cells (43). Such allografts are ultimately rejected in their model.

Together, the results of our mixed leukocyte reaction and flow cytometry studies suggest that DAC promotes a more generalized transition to an anti-inflammatory phenotype in CD8+ T cells than in CD4+ T cells. Targeted prevention of critical CD4+ T cell cytokine production combined with interrupted production of redundant CD8+ T cell cytokines may mediate DAC's beneficial effects in murine lung transplantation. This is consistent with previous findings that the presence of host CD4+ T cells prevents tolerance, but that the isolated removal of host CD4+ T cells prevents neither acute rejection nor CD28/B7 co-stimulation blockade mediated tolerance in this model (44). In that study, the predominant measured inflammatory cytokine present in graft-infiltrating CD4+ T cells of rejecting lungs was IFN-*γ*. In our study, allografts from DAC-treated hosts demonstrated a drop in the Th1 cytokine IFN-*γ* observed in both CD4 + and CD8+ T cells from allografts, as well as decreases in IL-17 and TNF-α production from CD8+ T cells not observed in CD4+ T cells. We have previously shown that IL-17 from CD8+ T cells mediates injury in allografts from hosts with both Th1 and co-stimulatory pathways blocked as a result of T-bet deficiency and anti-CD154 Ab treatment, respectively (45). Interruption of the TNF-α pathway, through either its removal by adsorption using extracorporeal hemoperfusion (46), or by inhibiting its conversion to its bioactive soluble form (47), improves allograft function following lung transplantation. TNF-α has also been associated with primary graft dysfunction following lung transplantation (48). The time course of our MLR, which evaluated early cytokine production in primed T cells within 24 h of exposure to stimulating allogenic cells, suggests that DAC treatment of hosts could conceivably block this as well.

Our model of FoxP3 depletion does not appear to directly injure the lung. Thus, allografts from dT treated WT hosts did not show worse injury than those from WT hosts not exposed to dT. Furthermore, host native lungs of DMSO-treated CD4 + FoxP3+ Treg-sufficient and -depleted hosts demonstrate similar histology. It is interesting that the combination of CD4 + FoxP3+ Treg-depletion and treatment with DAC can completely prevent collateral injury to the native lung of host mice. This unanticipated observation in our findings suggests a protective effect of DAC that is at least in part not dependent on CD4 + FoxP3+ T cells. However, an injurious component of the rejection process that is dependent on CD4 + FoxP3+ T cells cannot be excluded.

DAC is presented here in a model demonstrating its capacity to interrupt rejection. One could alternatively envision utilizing DAC in combination with induction protocols involving T reg infusion therapy (49). In this regard, the T reg-promoting capacity of DAC may facilitate ex-vivo T reg expansion and allow expedited tapering of conventional immunotherapies, thereby reducing individual and total immunosuppression dosing/toxicity.

Our manuscript is heavily observational and based on a flow cytometry approach. Cell culture, loss-off-function studies, and histologic evaluation supporting the flow cytometric findings were also employed. A more extensive evaluation using gain-of-function studies or exploration of the epigenetic factors by which DAC may influence CD8+ T cells (6, 50) was beyond the scope of our study.

Our study indicates that DAC initiated 72 h following lung allograft implantation disrupts acute lung allograft rejection in mice. This requires CD4 + FoxP3+ Tregs for maximal benefit and has marked effects on lung allograft T cell numbers, phenotype, and function. We propose DAC as a novel therapeutic target for acute lung allograft rejection.

## Data Availability

The raw data supporting the conclusions of this article will be made available by the authors, without undue reservation.
