# Supplementary figures and images for: Hypomethylating therapy mitigates acute allograft rejection in a murine lung transplant model
Source: Front Transplant. 2025 Jun 23;4:1612523. doi: 10.3389/frtra.2025.1612523 (PMC12230056; doi:10.3389/frtra.2025.1612523)

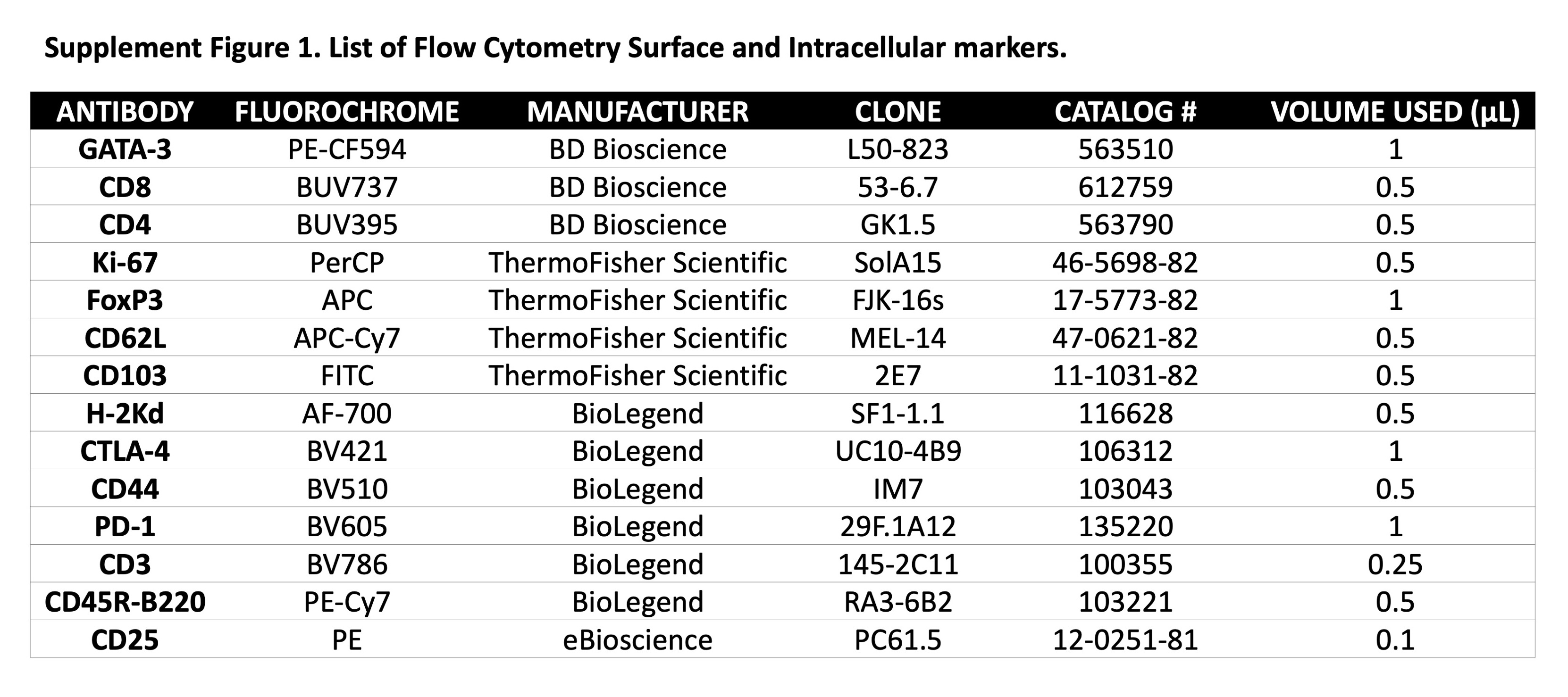

Supplement: Supplementary file 1 [file Image1.jpeg]

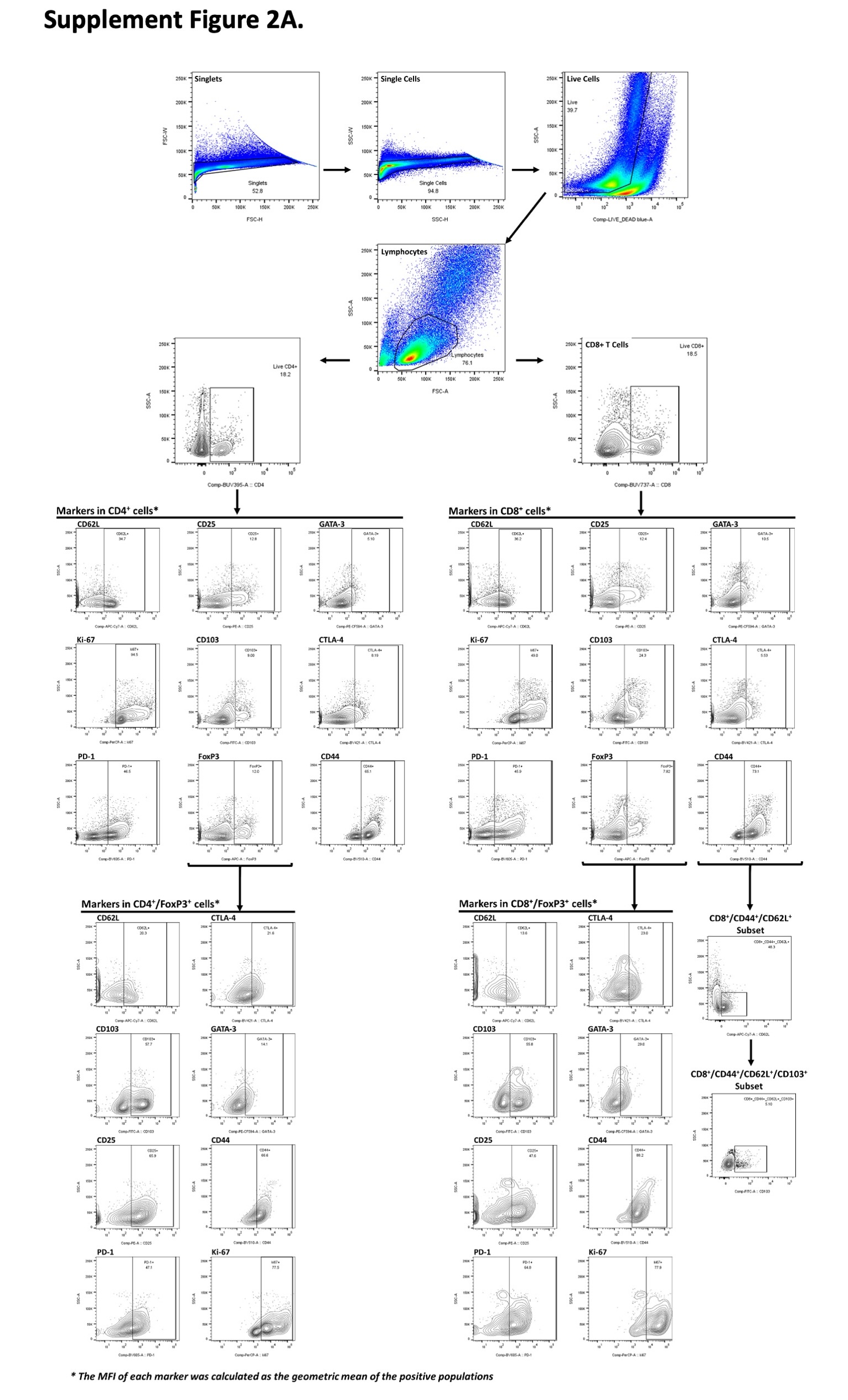

Supplement: Supplementary file 2 [file Image8.jpeg]

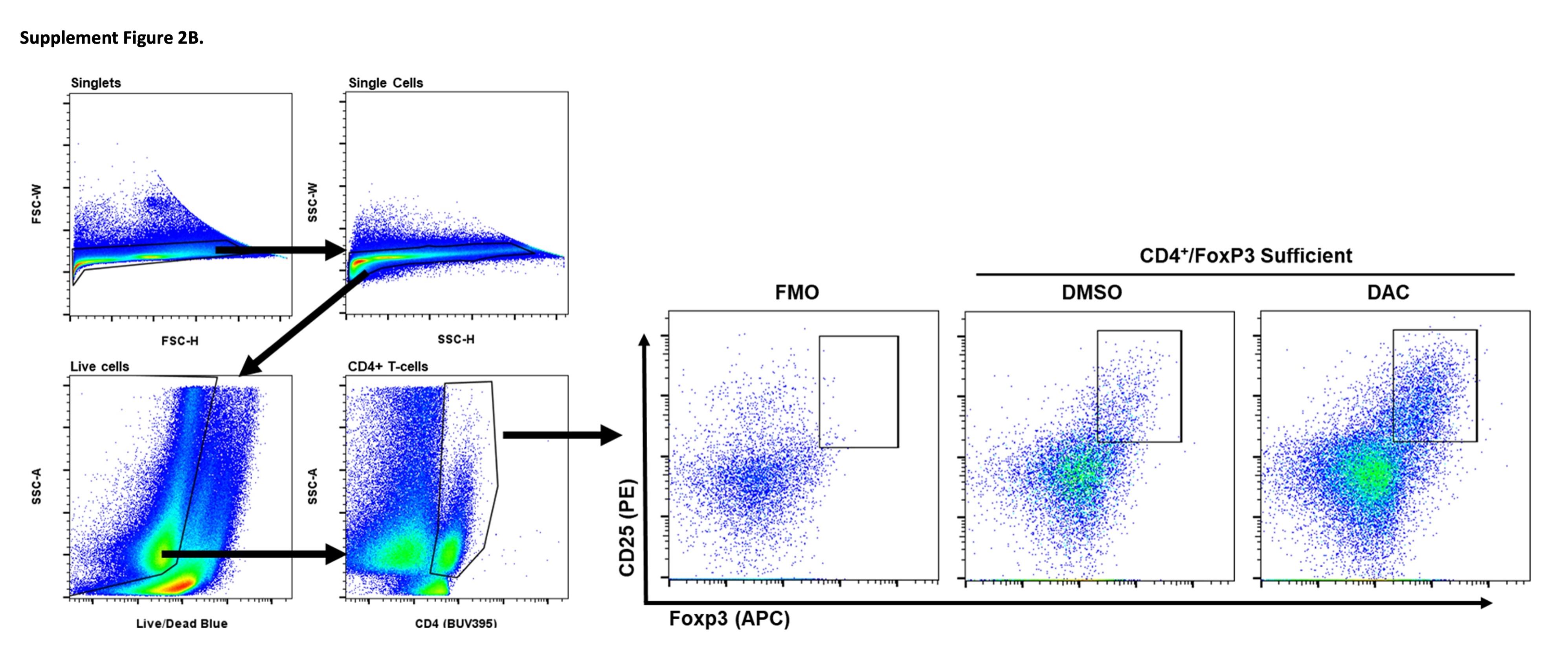

Supplement: Supplementary file 3 [file Image9.jpeg]

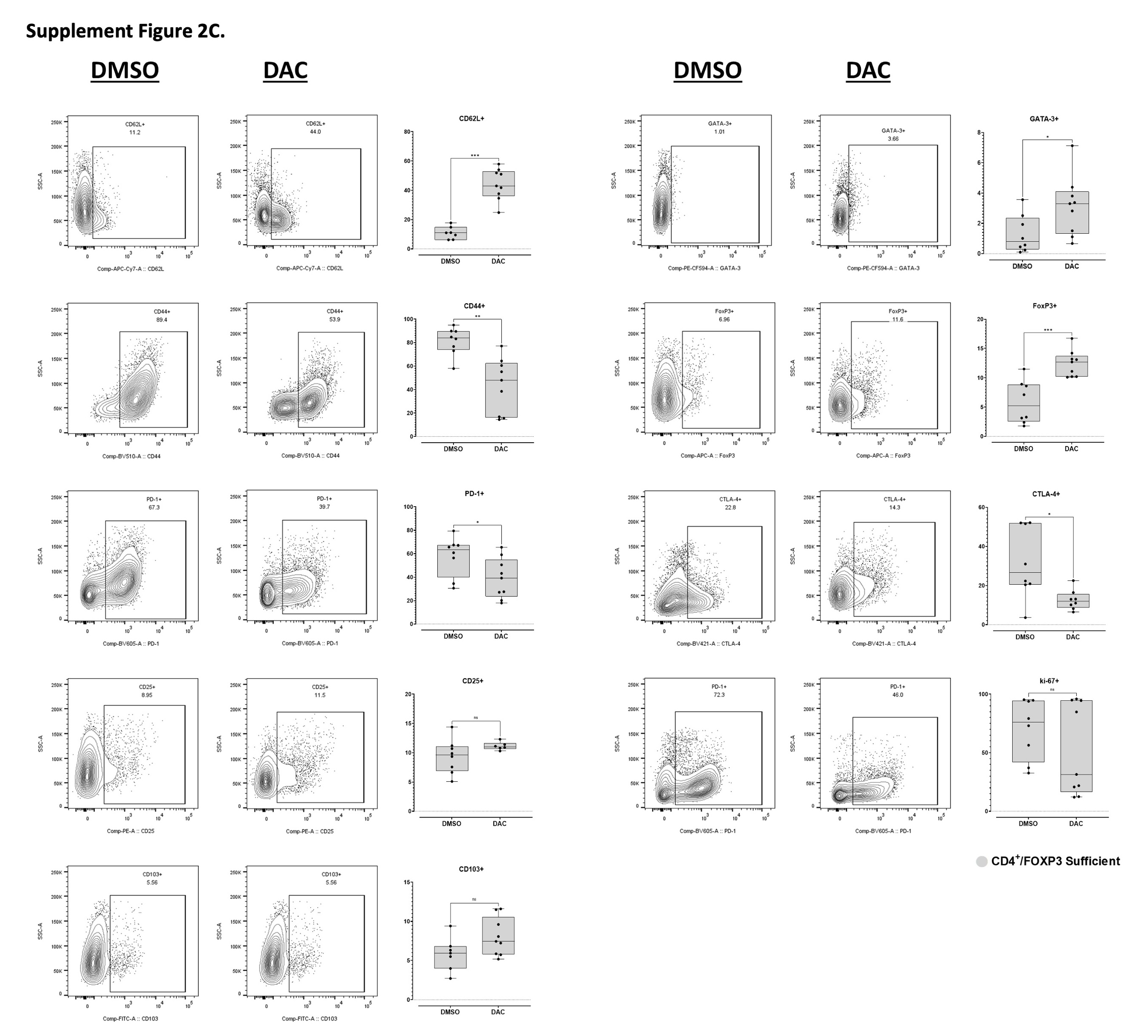

Supplement: Supplementary file 4 [file Image10.jpeg]

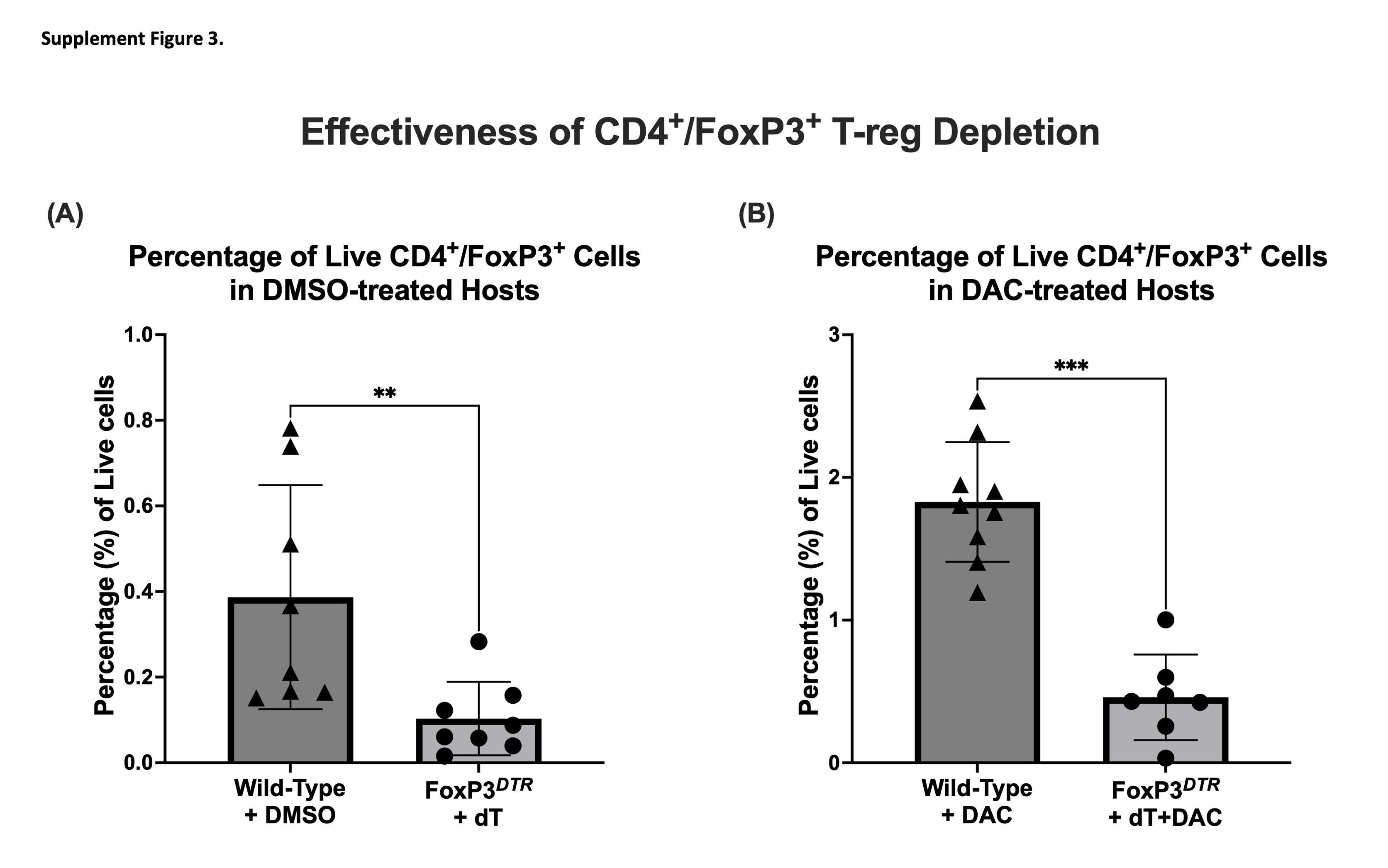

Supplement: Supplementary file 5 [file Image11.jpeg]

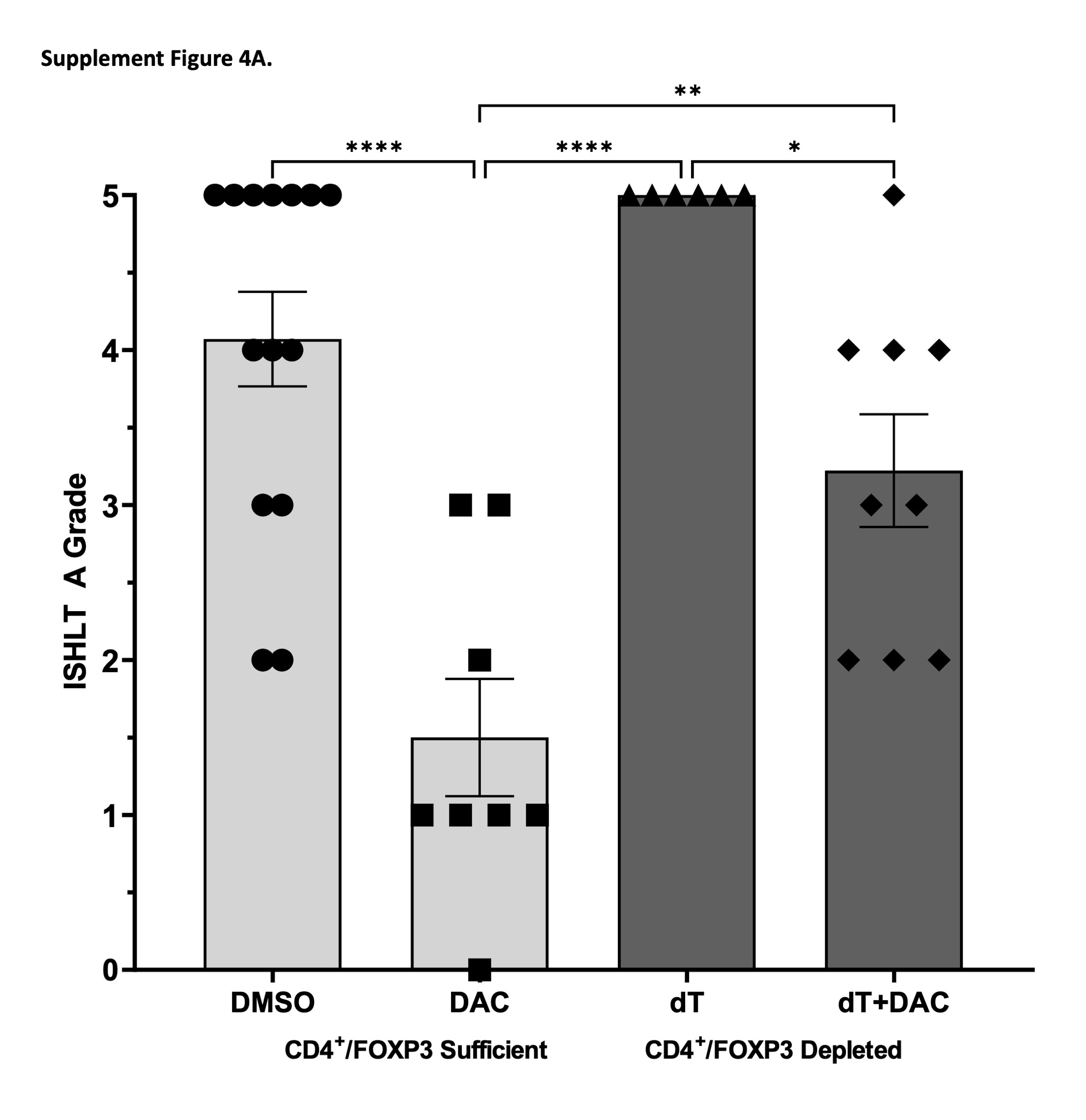

Supplement: Supplementary file 6 [file Image12.jpeg]

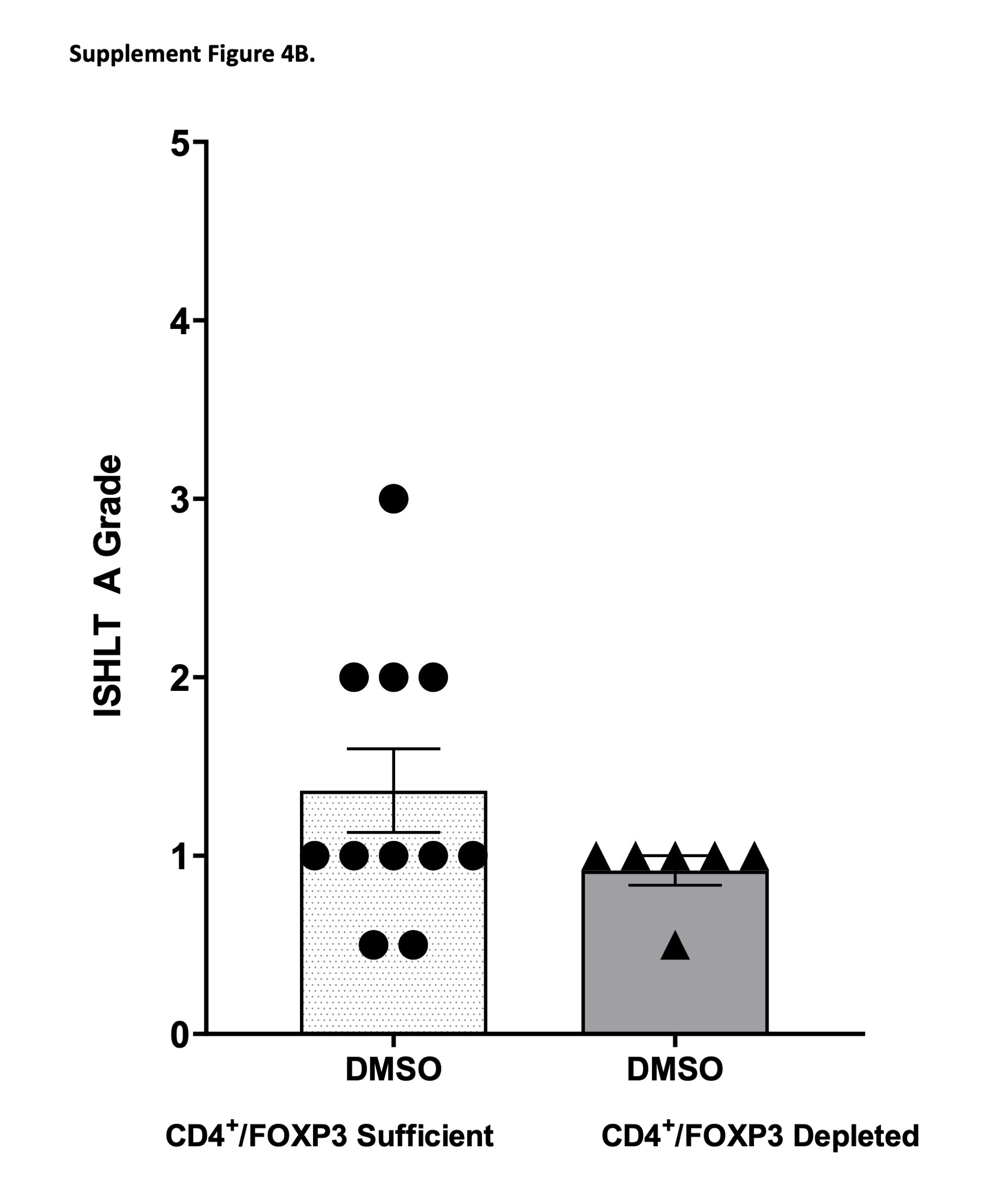

Supplement: Supplementary file 7 [file Image13.jpeg]

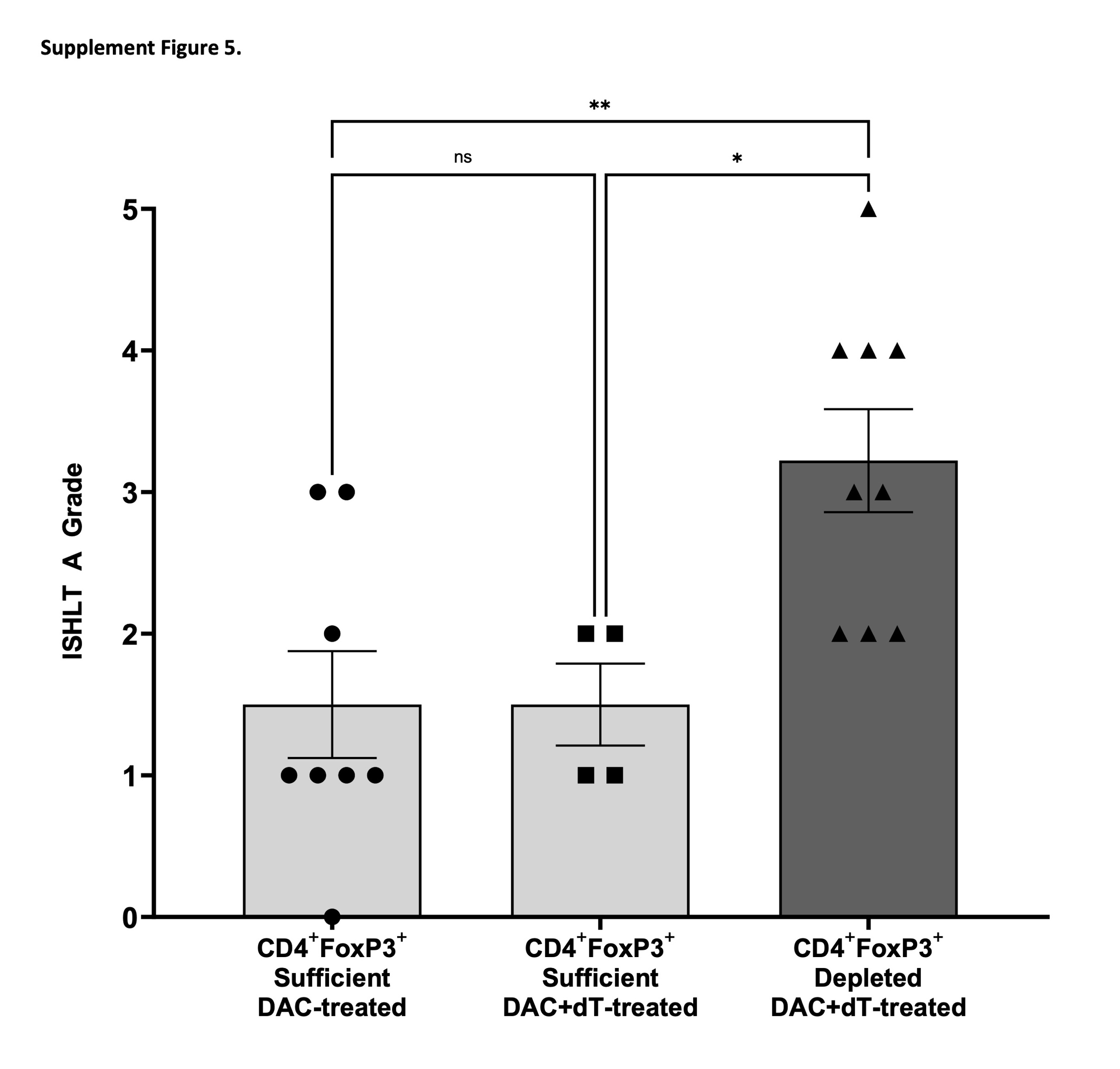

Supplement: Supplementary file 8 [file Image14.jpeg]
