# Supplementary material for: Hypomethylating therapy mitigates acute allograft rejection in a murine lung transplant model
Source: Front Transplant. 2025 Jun 23;4:1612523. doi: 10.3389/frtra.2025.1612523 (PMC12230056; doi:10.3389/frtra.2025.1612523)
Supplement: Supplementary file 9 [file Table2.docx]

Supplementary Material

# Supplementary Figures

**Supplement Figure 1.**

Table depicting the list of flow cytometry surface and intracellular markers specifying antibody, fluorochrome, manufacturer, clone, catalog number (#), and volume (µL) used per every 1x10^6^ cells stained.

**Supplementary Figure 2A. Flow cytometry gating strategy for identification of T cell subsets in lung allografts.**

Representative gating strategy used to analyze lung allograft-derived T cells. Singlets and live lymphocytes were gated, followed by the selection of CD4⁺ and CD8⁺ T cells. Expression of functional markers, including CD62L, CD25, GATA-3, Ki-67, CD103, CTLA-4, PD-1, FoxP3, and CD44, was assessed in CD4⁺, CD8⁺, CD4⁺FoxP3⁺, and CD8⁺FoxP3⁺ subsets. Specific CD8⁺ subpopulations, including CD8⁺CD44⁺CD62L⁺ and CD8⁺CD44⁺CD62L⁺CD103⁺, were further analyzed. MFI was calculated as the geometric mean fluorescence intensity of positive populations.

**Supplementary Figure 2B. FoxP3 fluorescence-minus-one (FMO) control and representative gating of CD4⁺FoxP3⁺ T cells.**

Gating strategy used to identify CD4⁺FoxP3⁺ T cells in lung allografts. The FMO control (left) was used to define the FoxP3⁺ population. Representative plots from DMSO- (middle) and DAC-treated (right) hosts demonstrate differences in FoxP3 and CD25 expression among CD4⁺ T cells.

**Supplementary Figure 2C.** **Sample Volcano Plot creation.**

Analysis flow to create volcano plots demonstrating the effect on the quantity of live allograft CD4⁺ T cells expressing various markers as a result of host treatment with either DAC or DMSO. Representative flow gates and box-and-whiskers plots of live allograft CD4⁺ T cells from CD4⁺FoxP3⁺ Treg-sufficient hosts expressing key functional markers (CD62L, CD44, PD-1, CD25, CD103, GATA-3, FoxP3, CTLA-4, and Ki-67). Hosts treated with DMSO or DAC. Cell quantity is expressed as a percentage (%) of total live allograft CD4⁺ T cells expressing a marker. Flow gate results from like treatments used to construct box and whiskers plots. Quantity differences between treatment groups, expressed as fold changes, were used to create volcano plots portraying the effect of DAC- vs DMSO- treatment of hosts on marker expression in live allograft CD4⁺ T cells.

**Supplement Figure 3.**

**Depletion of CD4^+^/FoxP3^+^ Treg by Diphtheria Toxin (dT). (A)** dT treatment of FoxP3*^DTR^* hosts (Vs DMSO treatment of Wild-Type hosts; POD 3, 5, and 7) effectively depletes CD4^+^/FoxP3^+^ T reg cells and **(B)** prevents the DAC-related increases in CD4^+^/FoxP3^+^ T reg cells. Data are presented as mean ± SEM (*N* = 7 - 9 per group). *P* ≤ 0.05 (**), P ≤ 0.01 (**), P ≤ 0.001 (****), and *P* ≤ 0.0001 (****); ns = not significant.

**Supplementary Figure 4A**

Histogram comparing lung allograft rejection scores using the International Society of Heart and Lung Transplantation (ISHLT) Grade A. Data are presented as mean ± SEM (N = 6–14 per group). P ≤ 0.05 (*), P ≤ 0.01 (**), P ≤ 0.001 (***), and P ≤ 0.0001 (****); ns = not significant

**Supplementary Figure 4B**

Supplement showing ISHLT grade for native lungs of CD4⁺FoxP3⁺ Treg- sufficient and –deficient hosts treated with DMSO Data are presented as mean ± SEM (N = 6–14 per group). P ≤ 0.05 (*), P ≤ 0.01 (**), P ≤ 0.001 (***), and P ≤ 0.0001 (****); ns = not significant.

**Supplementary Figure 5.**

Histogram showing ISHLT grade for allograft lungs of C57BL/6 wild-type mice (CD4⁺FoxP3⁺ Treg-sufficient host) treated with DAC or DAC+dT versus CD4⁺FoxP3⁺ Treg-depleted hosts treated with DAC+dT (n = 4-9 per group). Data are presented as mean ± SEM (N = 6–14 per group). P ≤ 0.05 (*), P ≤ 0.01 (**), P ≤ 0.001 (***), and P ≤ 0.0001 (****); ns = not significant
